# Long‐term reprogramming of primed microglia after moderate inhibition of CSF1R signaling

**DOI:** 10.1002/glia.24627

**Published:** 2024-10-24

**Authors:** Ana León‐Rodríguez, Jesús M. Grondona, Sonia Marín‐Wong, Manuel F. López‐Aranda, María D. López‐Ávalos

**Affiliations:** ^1^ Departamento de Biología Celular, Genética y Fisiología, Facultad de Ciencias Universidad de Málaga Málaga Spain; ^2^ Instituto de Investigación Biomédica de Málaga‐IBIMA Plataforma Bionand Málaga Spain

**Keywords:** CSF1R, microglia, neuroinflammation, PLX5622, priming

## Abstract

In acute neuroinflammation, microglia activate transiently, and return to a resting state later on. However, they may retain immune memory of such event, namely priming. Primed microglia are more sensitive to new stimuli and develop exacerbated responses, representing a risk factor for neurological disorders with an inflammatory component. Strategies to control the hyperactivation of microglia are, hence, of great interest. The receptor for colony stimulating factor 1 (CSF1R), expressed in myeloid cells, is essential for microglia viability, so its blockade with specific inhibitors (e.g. PLX5622) results in significant depletion of microglial population. Interestingly, upon inhibitor withdrawal, new naïve microglia repopulate the brain. Depletion‐repopulation has been proposed as a strategy to reprogram microglia. However, substantial elimination of microglia is inadvisable in human therapy. To overcome such drawback, we aimed to reprogram long‐term primed microglia by CSF1R partial inhibition. Microglial priming was induced in mice by acute neuroinflammation, provoked by intracerebroventricular injection of neuraminidase. After 3‐weeks recovery, low‐dose PLX5622 treatment was administrated for 12 days, followed by a withdrawal period of 7 weeks. Twelve hours before euthanasia, mice received a peripheral lipopolysaccharide (LPS) immune challenge, and the subsequent microglial inflammatory response was evaluated. PLX5622 provoked a 40%–50% decrease in microglial population, but basal levels were restored 7 weeks later. In the brain regions studied, hippocampus and hypothalamus, LPS induced enhanced microgliosis and inflammatory activation in neuraminidase‐injected mice, while PLX5622 treatment prevented these changes. Our results suggest that PLX5622 used at low doses reverts microglial priming and, remarkably, prevents broad microglial depletion.

## Main Points

- Neuraminidase‐induced neuroinflammation triggers long‐term microglial priming.
- Moderate CSF1R inhibition reverts microglial priming to a homeostatic phenotype, with only a partial and transient depletion of the microglial population.

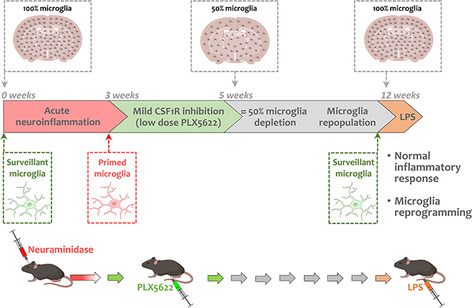

## 1 INTRODUCTION

In the last decades, microglia have been at the focus of increasing investigations, because of their unveiled participation in various physiological processes, and also for their demonstrated involvement in a variety of neurological diseases (extensively reviewed in Colonna & Butovsky, 2017; Gomez‐Nicola & Perry, 2014; Kettenmann et al., 2011; Loane & Kumar, 2016; Prinz et al., 2021; Tay et al., 2018; Tremblay et al., 2011; Watkins & Andrews, 2016; Yirmiya et al., 2015; among others). The role of microglia in neuroinflammation has been largely known, and as such they are widely recognized as brain macrophages in charge of orchestrating inflammation and immune responses within the brain (DiSabato et al., 2016; McGeer & McGeer, 1995; Prinz et al., 2021). Microglia pro‐inflammatory activation can be triggered by pathogens, traumatisms or any other cue indicative of the loss of homeostasis in the nervous system milieu. Then, resident microglia and astrocytes activate, the blood–brain barrier permeabilizes, peripheral immune cells are recruited, and a plethora of inflammatory mediators are secreted in a timely fashion, in a process that peaks in a few days, and recedes to basal levels later on, along with the restoration of homeostasis (reviewed in Estes & McAllister, 2014; Prinz & Priller, 2014; Ransohoff, 2016; Ransohoff & Cardona, 2010). However, it has been recently described that, after undergoing activation, microglia may stay chronically activated or, in other cases, may deactivate but retain memory of the past inflammatory event, a status known as primed microglia (Neher & Cunningham, 2019; Norden & Godbout, 2013; Perry & Teeling, 2013; Purisai et al., 2007; Wendeln et al., 2018).

Primed microglia are more sensitive and hyperreactive to new inflammatory stimuli, and have been suggested to trigger or aggravate neurodegenerative diseases (De Sousa et al., 2021; Norden et al., 2015) and cognitive impairment (Muccigrosso et al., 2016), to be involved in behavioral sensitization (Frank et al., 2020; Weber et al., 2019), to be responsible for psychiatric disorders (Fenn et al., 2014; Lisboa et al., 2016), or to be related to energy balance disturbances (Fernandez‐Arjona et al., 2022a; Reis et al., 2015; Spencer et al., 2019; Valdearcos et al., 2017), among many other alterations. Furthermore, the primed status of microglia may endure for months, as has been demonstrated in rodents (Fernandez‐Arjona et al., 2022a; Fernandez‐Arjona et al., 2022b; Wendeln et al., 2018) or maybe even years, thus representing a silent risk factor for neurological disorders. In such scenario, knowing the physiology of microglia and strategies for their control are of increasing interest.

The receptor of the colony stimulating factor 1 (CSF1R) is one of the therapeutic target molecules expressed by microglia that has raised ample attention (reviewed in Chitu et al., 2016). It is expressed not only by microglia, but also by other myeloid cells such as macrophages, monocytes and osteoclasts (Dai et al., 2002; Erblich et al., 2011; Raivich et al., 1998; Sasmono et al., 2003; Stanley & Chitu, 2014). In fact, disruption of the CSF1R gene in mouse resulted in osteopetrosis (due to osteoclast dysfunction) and mononuclear phagocyte deficiency (Dai et al., 2002). Besides, mutations in this receptor in humans cause hereditary diffuse leukoencephalopathy, a central nervous system disease with behavioral and psychiatric symptoms (Hume et al., 2020; Rademakers et al., 2011).

Various CSF1R inhibitors have been recently developed to target CSF1R signaling, fueled by their therapeutic potential in cancer treatments (reviewed in Cannarile et al., 2017). Many of these inhibitors are small molecules that penetrates the blood–brain barrier, thus targeting CSF1R‐expressing brain cells. As CSF1R signaling is required for cell differentiation, proliferation and survival, some of these inhibitors provoke a significant reduction of microglia population in the brain (Elmore et al., 2014; Pyonteck et al., 2013), along with some derangement of peripheral immune cells (Funk & Klein, 2019; Lei et al., 2020; Sanchez et al., 2021; Spiteri et al., 2022) which has, on the other hand, been the subject of discrepancies among author. Inhibitor dose and treatment duration may explain these disagreements, with the additional contribution of the higher sensitivity of microglia to CSF1R inhibition compared to peripheral myeloid cells (Okojie et al., 2023; Wheeler et al., 2018). Thus, this family of small molecule inhibitors has emerged as very potent tools for deciphering microglial role in the healthy brain, with PLX3397 and its more specific and brain penetrant partner PLX5622 (Spangenberg et al., 2019) among the most widely used. These compounds are tyrosine kinase inhibitors with the highly valued property of penetrating the brain easily (Butowski et al., 2016). Remarkably, as soon as the exposure to the PLX compound is interrupted, microglia repopulate the brain, mostly by the active proliferation of a residual surviving microglial fraction (Elmore et al., 2014; Huang et al., 2018; Najafi et al., 2018; Zhou et al., 2022). Thus, CSF1R inhibition by PLX compounds is reversible, which represents a highly attractive feature of these compounds. Intriguingly, the restored population of microglial cells seems to present a surveillant phenotype, that is, they appear and behave more like naïve cells than as activated or primed ones, which has led to suggest an immunomodulatory capacity of these PLX compounds (Elmore et al., 2014; Rice et al., 2017; Shi et al., 2022; Willis et al., 2020).

In spite of its promising therapeutic potential, CSF1R inhibition has also raised some concerns. First, the almost total ablation of microglia from the brain represents a highly vulnerable situation, particularly in the case of microbial infections (Funk & Klein, 2019; Seitz et al., 2018; Wheeler et al., 2018), precluding the translation of the use of these inhibitors to the clinic. Even so, their therapeutic potential in cancer treatments has been intensely explored (reviewed in Cannarile et al., 2017; Wen et al., 2023), and PLX3397 is already approved by the FDA for a particular type of tumor (tenosynovial giant cell tumor). Another drawback is related to the impact of CSF1R inhibition on peripheral immune cells, as the receptor is expressed in cells of the myeloid lineage. Although evidence points to a moderate impact on peripheral cells compared to that observed in microglia (Bray et al., 2022; Spangenberg et al., 2019), some authors raised serious concerns after describing significant disturbances in peripheral immune cells even involving non‐myeloid cells (Green & Hume, 2021; Lei et al., 2020).

In any case, because of the promising therapeutic potential of CSF1R inhibitors, and in particular the PLX5622 compound, we aimed to investigate the capacity of PLX5622 to revert the primed phenotype of microglia, but using a moderate dose that prevents a complete ablation of this cell population. A model of acute severe neuroinflammation triggered by the intracerebroventricular (ICV) administration of neuraminidase (NA) in mice was used to induce long‐term microglial priming (Fernandez‐Arjona et al., 2022a, 2022b). A low‐dose treatment with PLX5622 was followed by a prolonged withdrawal period, after which microglia response was addressed by peripheral immune stimulation with lipopolysaccharide (LPS). Our results demonstrate that PLX5622 is able to switch the phenotype of primed microglia to a naïve one, thus supporting the immunomodulatory capacity of PLX5622 used at low doses, independently of the undesirable broad depletion of microglia.

## 2 METHODS

### 2.1 Animals

**FIGURE 1:**
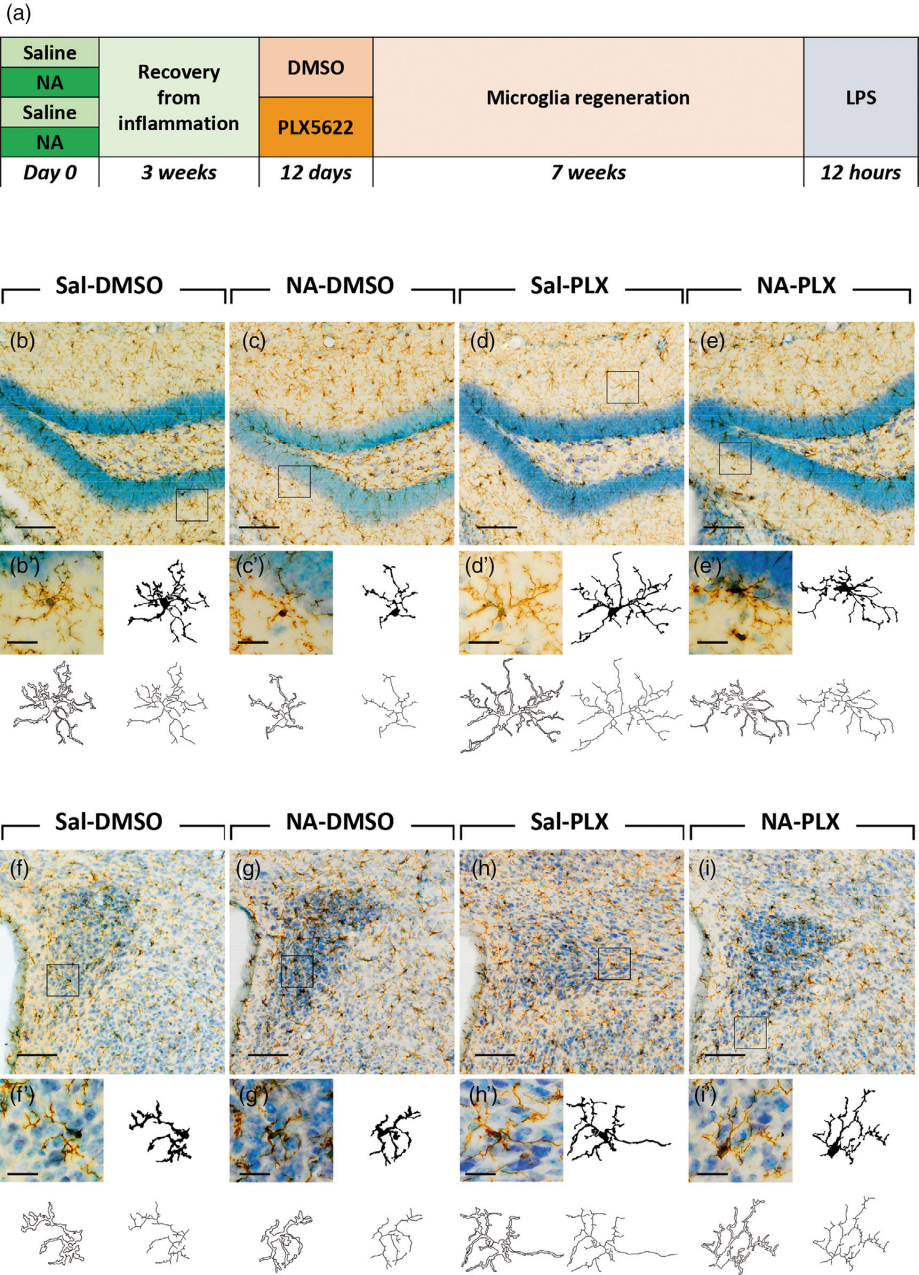
Long‐term impact on microglia of a transient low‐dose PLX5622 treatment applied after acute neuroinflammation in mice. (a) Scheme of the experimental design, where mice were intracerebroventricular (ICV) injected with neuraminidase (control mice were injected with saline) to induce neuroinflammation and microglial priming. Three weeks later, mice received a daily IP dose (60 mg/kg) of PLX5622 (control mice were injected with vehicle DMSO). Seven weeks after treatment completion (12 weeks from the initial ICV injection), all mice received an intraperitoneal (IP) dose of lipopolysaccharide (LPS) to induce peripheral inflammation, and were euthanized 12 h later. (b–i) IBA1 immunostaining of representative brain sections of mice from the above experiment. Microglia were studied in the dentate gyrus of the hippocampus (b–e) and in the paraventricular nucleus of the hypothalamus (f–i). In each image, a representative cell (squared) is shown enlarged below (b' through i', respectively), along with its profiles (filled, outlined, and skeleton shapes) obtained for the subsequent morphological analysis. Scale bars in b‐i are 100 μm. Scale bars in b'–i' sampled cells are 20 μm. DMSO, dimethyl sulfoxide; LPS, lipopolysaccharide; NA, neuraminidase; PLX, PLX5622; Sal, saline.

Male mice of the strain C57BL/6J and 3 months old (≈25 g) were used (Charles River, France). Mice were housed under standard conditions: 12 h light/dark cycle, 23°C and 60% humidity, with food and water available ad libitum. Experiments were carried out following the Spanish legislation (RD 53/2013) and the European Union regulation (Directive 2010/63/EU), with the approval of the ethics committee of Universidad de Málaga and Consejería de Agricultura, Ganadería, Pesca y Desarrollo Sostenible, Junta de Andalucía (Ref. 04/10/2018/145). The animals underwent an experimental design that involved an ICV injection of NA or its vehicle, followed by the intraperitoneal (IP) administration of PLX5622 compound during 12 days, and finally an IP injection with LPS 12 h prior to euthanize by transcardiac perfusion. *N* = 15 mice were assigned to each experimental group. The timetable of this experiment is summarized in the scheme of Figure 1a.

### 2.2 ICV injection

Mice were subjected to an ICV injection of NA to provoke acute neuroinflammation. They were first anesthetized with a mix of ketamine (60 mg/kg) and xylazine (10 mg/kg), and placed in a stereotaxic frame. The skin covering the skull was shaved and cut along the sagittal midline with a scalpel to expose the skull. Once located the Bregma point, a hole was made in the skull using a drill in coordinates 0.9 mm lateral and 0.1 mm posterior from Bregma (Franklin & Paxinos, 2008). The needle was then lowered to 2.0 mm below the dura mater surface to reach the right lateral ventricle. The solution was injected with the aid of a pump at a rate of 0.2 μL/min during 10 min. NA‐treated mice were injected with 50 mU (2 μL) of NA from *Clostridium perfringens* (Sigma‐Aldrich, N3001) dissolved in 0.9% sterile saline, while control mice received the same volume of the vehicle. Once finished the infusion the skin was sutured with resorbable thread and cleaned with povidone. The recovery of animals from anesthesia, as well as signs of pain, were monitored during the following hour.

### 2.3 Treatment with PLX5622

The CSF1R inhibitor PLX5622 (PLX5622 hemifumarate; MedChemExpress reference HY‐114153A, batch 57346) was dissolved in dimethyl sulfoxide (DMSO) to a final concentration of 100 mg/mL; solubilization was aided by sonication (three pulses of 30 s in ice). Three weeks after the ICV injection of NA, mice were treated with PLX5622 or with an equivalent volume of DMSO. The treatment consisted in one daily IP injection of PLX5622 at a dose of 60 mg/kg (1.8 mg/mice of ≈30 g body weight, dissolved in 18 μL of DMSO) during 12 days. Once the treatment was completed, mice were left undisturbed for 7 weeks.

### 2.4 LPS stimulation and tissue sampling

Seven weeks after PLX5622 treatment termination the mice received a second inflammatory stimulus, in this case consistent in 1 mg/kg of LPS from *Escherichia coli* (Sigma‐Aldrich, L2630) administered IP. Twelve hours later the animals were euthanized. After being anesthetized as before, a blood sample was taken from the heart and the mice were transcardially perfused following two different protocols according to the subsequent destination of the brain tissue: (1) after perfusion with cold saline solution containing 10 IU/mL of heparin to clear the blood, the brain was frozen in dry ice; this tissue was used for RNA and protein extractions; (2) an initial perfusion with cold saline solution was followed by perfusion with 4% paraformaldehyde; the brain was dissected out, immersed in the same fixative at 4°C for 24 h, and washed with PBS. This tissue was intended for histologic studies.

Frozen brains were stored at −80°C until processing. For microdissection of the brain regions of interest, the brains were retrieved from the freezer and placed in dry ice. A mouse brain matrix (RWD, 800‐00143‐00) and high‐profile stainless‐steel microtome blades were also dry ice cold. Each brain was placed in the matrix and allowed to warm only until soft enough for the blade to cut across. Coronal 2 mm thick pieces were obtained and kept frozen. Pieces containing the regions of interest (rostral hippocampus and periventricular hypothalamus) were retrieved from the matrix and further dissected using a 1 mm tissue biopsy punch, maintaining the tissue frozen during all the procedure. The tissue dissected from the left side of the brain was placed in RNAse‐free tubes and was later processed for RNA isolation. The tissue dissected from the right side of the brain was placed in low protein binding tubes and processed for protein extraction.

### 2.5 Immunohistochemistry

The brains destined to histological studies were sectioned using a vibratome (Leica VT1000S). 40‐μm coronal sections were organized in series collections and stored in PBS. Immunostaining was carried out with rabbit anti‐IBA1 to label microglia (1:500; Wako 19‐19,741), or with rabbit anti‐GFAP (1:10000; Dako Z0334) to label astrocytes. First, to inactivate endogenous peroxidase, the sections were treated with a solution of 10% methanol and 3% hydrogen peroxide in PBS during 45 min. After washes with PBS, non‐specific binding sites were masked by pre‐treatment with a blocking solution (PBT: 0.3% bovine serum albumin, 0.3% Triton X‐100 in PBS pH 7.3). Primary antibodies were incubated overnight at 4°C. The next day sections were washed with PBS, and incubated in secondary biotinylated antibodies (1:1000; biotin‐conjugated goat anti‐rabbit, Pierce 31,820) at room temperature for 1.5 h. Afterwards, the avidin‐biotin‐complex (ABC) amplification system was used (1:250; ABC Thermo Fisher Scientific, ref. 32020); sections were incubated at room temperature for 45 min. Finally, peroxidase reaction was developed with a solution of 0.05% diaminobenzidine and 0.03% hydrogen peroxide in PBS during 10 min. Sections were then washed, mounted in gelatin‐treated slides and air‐dried overnight. Counterstaining of cell nuclei was done with 0.1% toluidine blue for 10 min; then sections were dehydrated and mounted with Eukitt mounting medium. Sections washes were done with PBS pH 7.3, and antibodies were diluted with PBT solution.

### 2.6 Cell counts on immunostained sections

Both microglia and astrocyte counts were carried out in two defined brain regions: the dentate gyrus (DG) of the hippocampus and the paraventricular nucleus (PVN) of the hypothalamus. The coronal sections used ranged between −1.5 and −2.0 mm from Bregma. These regions were scanned with an Olympus VS120 scanner microscope, using a UPLSAPO 20× objective. Cells were counted on the obtained images using the plugin Cell Counter for the free software FIJI (http://fiji.sc/Fiji). A total of 1 section for PVN, and 2 sections for DG per animal were counted, and the mean value obtained was expressed as number of cells/mm^2^.

### 2.7 Morphological analysis of microglial cells

IBA‐1 stained sections were used for the morphological evaluation of microglial cells in the same two regions indicated above, the DG and the PVN. For this purpose, in this case sections were scanned with an Olympus VS120 scanner microscope but using a higher resolution objective (UPLSAPO 60× oil immersion objective). To obtain highly detailed profiles of the ramifications of the cells, a multi‐plane virtual‐Z mode was used. Thus, from each histologic field, 20 images 1 μm apart spanning in the Z plane were captured and coupled, rendering detailed images of microglial cells. Then, from these larger images, individual cells were selected and cropped, following non‐biased criteria: (1) random selection, in the DG starting in the infrapyramidal blade towards the suprapyramidal blade, and in the PVN starting at the wall of the third ventricle and moving away towards the parenchyma; (2) trying to avoid overlapping with other nearby cells; and (3) with a full view of both the cell body and branches. For each region, DG and PVN, a total of *n* = 10 cells were selected from different sections and from *n* = 6–7 animals per experimental group.

The images of the selected cells were then processed for their morphometric analysis using the free software FIJI. For each cell image sequential steps were as follows: (1) Split color channels red, blue and green; as the blue channel enhances the IBA1 staining, this channel was used from now on. (2) Obtain a binary image, using a pre‐established threshold value in the grayscale, which was the same for all images. (3) Manual editing of the binary image, with the aim of filling gaps in branches and erasing debris from other cells; this task was performed in view of the original color image. (4) Get three parallel images of the cell: a filled shape, an outlined shape, and a skeleton shape. These three images were afterward used to obtain morphological parameters.

For a more comprehensive characterization of cellular morphology, three different methods for morphological evaluation were used: Fractal analysis, Skeleton analysis and Sholl analysis.

We conducted the Fractal analysis using the FracLac plugin for ImageJ (Karperien A., version 2.5), utilizing both the filled and outlined shapes of each cell. This yielded 15 parameters including *Cell area*, *Cell perimeter*, *Lacunarity*, *Fractality*, *Circularity*, *Convex hull area*, *Convex hull perimeter*, *Convex hull circularity*, *Density*, *Roughness*, *Span ratio*, *Bounding circle diameter*, *Maximum span across the convex hull*, *The ratio of maximum/minimum convex hull radii* and *Mean radius*. These parameters are further explained in Fernandez‐Arjona et al. (2017).

Skeleton analysis was made with the free plugin *AnalyzeSkeleton* plugin for ImageJ (Arganda‐Carreras I., version 3.4.2) (Arganda‐Carreras et al., 2010) using the skeletonized shape of each cell. The center of the cell, which corresponds to the soma, was manually selected and nine parameters were directly obtained: *Branches* (the total number of branches of each cell), *Junctions* (the total number of branch intersections), *End‐point voxels* (the number of voxels, or 3D pixels, that have less than two neighbor voxels), *Junction voxels* (the number of voxels that have more than two neighbor voxels), *Slab voxels* (the number of voxels that have exactly two neighbor voxels), *Average branch length* (the mean length of the branches in a cell), *Maximum branch length* (the maximum length of all the branches in a cell), *Triple points* (the number of junctions in the cell that join three different branches), and *Quadruple points* (the number of junctions in the cell that join four different branches).

Finally, Sholl analysis was carried out using the free plugin *SNT* plugin for ImageJ (Longair M., version 4.3.0‐pre‐release3) (Longair et al., 2011) using the skeletonized shape of each cell. This method, based on the traditional Sholl approach, involves creating concentric circles of increasing 0.12 μm diameter around the cell soma; then branch intersections with these circles are counted. As above, the soma was manually selected as the centroid of the circles. From this analysis, the number of branch intersections in each concentric circle (centroid distance) was represented; also, four new parameters were calculated for each microglial cell: *Critical radius* (the radius of the circle with the highest number of intersections), *Process maximum* (the highest number of intersections at any of the circles), *Primary branches* (the number of intersections occurring in the smallest circle), and *Ramification index* (obtained by dividing the *Process maximum* by the *Primary branches*).

### 2.8 RNA isolation and quantitative polymerase chain reaction

RNAzol (Molecular Research Center Inc.) was used for total RNA isolation. A volume of the reagent (0.5 mL/50 mg of tissue) was added to the still frozen tissue. Once thawed, tissue disruption was done mechanically with the aid of the pipette tip and a plastic micro pestle. The rest of isolation was performed according to the reagent's indications. The pellet of RNA was dissolved in 30 μL of RNAse‐free water, and then stored at −80°C. RNA concentration was measured in a Nanodrop equipment.

Reverse transcription (RT) of RNA samples to obtain cDNA was carried out with the PrimeScript RT Master Mix reaction mixture (Takara, RR036A). RNA samples were taken to a concentration of 500 ng/μL with RNAse‐free water. Each RT reaction tube contained 8 μL of the RNA sample (4000 ng of RNA) and 2 μL of the RT reagent, resulting in a final reaction volume of 10 μL. The RT reaction was carried out at 37°C during 15 min, followed by inactivation of the enzyme at 85°C for 5 min. Finally, a 1:10 dilution was done with water, thus obtaining a final concentration of 5 ng of cDNA equivalent/μL. cDNA samples were stored at −20°C.

To measure the expression level of genes of interest, quantitative polymerase chain reaction (qPCR) was then performed with these cDNA samples. The genes of interest were identified by searching their sequences in the Genbank NCBI Reference Sequence database. Primers were designed using the Primer Blast program available at https://blast.ncbi.nlm.nih.gov, and are listed in Table S1. Quantification of the target mRNA levels in the samples was performed using real‐time PCR with SYBR Green I fluorescent dye. For this, the hot start reaction mix FastStart Essential DNA Green Master (Roche 06402712001) was employed. The reaction mix (10 μL final volume) contained 5 μL of master mix, 1 μL of each forward and reverse primers (at a concentration of 0.4 μM each), and 4 μL of cDNA (20 ng of cDNA/μL). Two or three replicates per animal and gene were done. The PCR reaction was conducted using a LightCycler® 96 instrument (Roche). Amplification curves, dissociation curves, and quantification cycles (Cq) were obtained for each target gene. Data analysis was performed using the software provided with the LightCycler® 96 instrument.

Prior to running the qPCR with the samples, PCR efficiency (*E*) was estimated by amplifying serial dilutions of the cDNA samples. *E* was calculated using the equation *E* = 10^[−1/slope]^ − 1. The expression level of the target genes was normalized to the expression of a reference gene, glyceraldehyde 3‐phosphate dehydrogenase (GAPDH). The relative expression of each target gene compared to GAPDH was calculated using the formula:

$$\text{Gene expression}\;\left(\text{relative to GAPDH}\right)={\left(E_{\text{targe}t}\right)}^{\mathrm{\Delta CP}\;\text{target}}/{\left(E_{\text{GAPDH}}\right)}^{\mathrm{\Delta CP}\;\text{GAPDH}},$$

where *E* represents the efficiency of the PCR reaction for each gene (both the target gene and the reference gene GAPDH), and ΔCP is the difference between the Cq values of the control sample and the experimental sample for both the target and the reference genes.

### 2.9 Protein extraction and multiplex immunoassay

Brain tissue samples were kept at −80°C until protein extraction, which was carried out using RIPA buffer (125 mM NaCl, 0.5% Triton X‐100, 1 mM EDTA, 1 mM EGTA, 20 mM HEPES pH 7) with the cOmplete™ Mini Protease Inhibitor Cocktail (Roche). Ice‐cold RIPA was added to tissue at a ratio of 150 μL/15–30 mg of tissue. The tissue was homogenized manually using a plastic micro pestle in microcentrifuge tubes. Then, two 5‐sec sonication steps (60–70 Hz) were applied to aid tissue and cell disruption, maintaining samples in ice during throughout the procedure. Samples were then centrifuged (14.000 g for 15 min at 4°C) and the supernatants recovered. The protein concentration in the extracts was determined with the Pierce™ bicinchoninic acid protein assay (Thermo Fisher Scientific). Protein concentration ranged 2–6 mg/mL.

These protein extracts were used for the quantification of cytokines by multiplex immunoassay with the ProcartaPlex™ Mouse High Sensitivity Panel 5plex (Invitrogen), which included five analytes: IFNγ, IL‐2, IL‐6 and TNFα. The assay was run following the manufacturer's indications by the proteomics facility of Universidad de Málaga. The results were read and processed in a Bioplex 200 equipment (Biorad). For each cytokine the results were normalized by the protein content of the sample.

### 2.10 Statistical analysis and principal components analysis

The statistical analysis of data was performed with SPSS Statistics® 24 (IBM®) and GraphPad Prism 9 software. First, the ROUT method for identifying outliers was employed to remove those values that were highlighted by the analysis. Subsequently, the Kolmogorov–Smirnov (*n* < 50) and Shapiro Wilk (*n* > 50) normality tests and the Levene homoscedasticity test were used to confirm if parametric methods were appropriate to our data sets. Then, one‐way analysis of variance (ANOVA) was carried out, followed by Tuckey post hoc test for pairwise comparisons between groups. Differences between groups were considered significant when the *p* value was <.05.

If the data did not meet the assumption of normality, a Kruskal–Wallis test for non‐parametric data was employed, followed by a Dunn's post hoc test for pairwise comparisons between groups. If the data met the assumption of normality but not the assumption of homoscedasticity or equality of variances, a Brown‐Forsythe and Welch ANOVA post hoc test was utilized for pairwise comparisons between groups. In either case, data were considered statistically different when the *p* value was <.05.

Histograms show the mean ± the standard deviation (SD). For morphological parameters, data distribution is presented as violin plot, through the estimated density calculated with the Kernel Density Estimation (KDE) method; violin plots are truncated at the maximum and minimum values of each dataset and displayed with a medium smoothing to show the overall distribution while preserving many details of the original data. In graphs, statistically significant differences between groups have been signposted with asterisks. For simplicity, less relevant comparisons have been omitted (namely Sal‐DMSO vs. NA‐PLX, NA‐DMSO vs. Sal‐PLX).

On the other hand, principal components analysis (PCA) was carried out with all 27 morphological parameters obtained from individual microglial cells. PCA was done for cells from DG and for cells from PVN separately. The goal was to explore the relationships among the microglial morphological parameters which yielded significant differences in the ANOVA analysis. PCA enables the reduction of the number of variables and their transformation into a new set of variables with the following characteristics: (1) the new variables are linear combinations of the original ones, (2) they are uncorrelated with each other, and (3) they can collectively explain a significant portion of the variance. To consider the results valid, the new variables (or factors) derived from PCA should explain over 60%–70% of the variance.

The analysis utilized the correlation matrix derived from the entire sample of animals (comprising 27 subjects ICV injected with either saline or NA and later treated with DMSO or PLX5622). The adequacy of the sample was assessed through the Bartlett sphericity and Kaiser‐Meyer‐Olkin (KMO) tests. Factors with eigenvalues exceeding 1 were maintained for further analysis. The significance of “factor loading” (i.e., the contribution of each variable to a factor) was determined to be greater than 0.05. Subsequently, given that factor scores represent the relative contribution of each loading pattern, ANOVA was employed, followed by Tuckey post hoc test for pairwise comparisons between animals injected with NA and with saline, and animals treated with PLX5622 or with DMSO.

## 3 RESULTS

### 3.1 Impact of low‐dose PLX5622 treatment followed by a prolonged withdrawal period and LPS challenge on microglia and astrocyte populations

Here we design an experimental paradigm aimed to explore the capacity of PLX5622 to revert microglial priming (Figure 1a). Microglial priming was provoked by the ICV administration of neuraminidase (NA) to mice, which we have previously shown that generates acute ventricular neuroinflammation (Granados‐Durán et al., 2015) and long‐term priming of microglia (Fernandez‐Arjona et al., 2022a, 2022b). The neuroinflammatory process peaks during the first week, and is largely solved after 3 weeks. Thus, to allow for the neuroinflammatory process to proceed until its completion, PLX5622 treatment was initiated 3 weeks after NA administration. PLX5622 treatment consisted in a daily IP dose of 60 mg/kg during 12 days, while control mice received an equivalent volume of the vehicle DMSO. This treatment protocol with PLX5622 marks a substantial difference compared to those used in the majority of works published to date, because the dose employed here is significantly lower (about half). In this way, we aimed not to deplete microglia, as most previous works intended, but to influence its phenotypic profile. Once PLX5622 treatment was completed, a restoration/repopulation period of 7 weeks was allowed, which is much longer than the time reported to be required for microglia repopulation. Thereby we pursued to evaluate the primed state of microglia at long‐term, as well as to guarantee a total restoration of microglial population. Finally, a second inflammatory stimulus, consisting in LPS peripherally administered, was applied to trigger microglial activation, whose response should be enhanced in the case of primed microglia. Thus, this represents an experimental paradigm of a double inflammatory insult with a long relapse time, adding the PLX5622 treatment in between.

Microglia were studied in two brain regions: the rostral hippocampus, which is relatively close to the lateral ventricles (the site of ICV administration of NA/saline), and the periventricular hypothalamus, because it is an area of influence of the ventricular inflammation driven by NA which, after ICV administration, disperses along the ventricular system. Representative images of the dentate gyrus (DG) of the hippocampus stained with IBA1 show that microglia cell density is similar in all experimental groups (Figure 1b–e), which is expected because of the long withdrawal period after PLX5622. A closer look to microglia morphology allows to notice that cells from NA‐treated mice are morphologically different (more intensely stained, with fewer and shorter ramifications) from microglia of other experimental groups (representative cells are shown in Figure 1b′–e′). Similarly, in the paraventricular nucleus (PVN) of the hypothalamus microglial cell density seems to be unaltered after NA injection and/or PLX5622 treatment (Figure 1f–i). However, cells appear with a different morphology and with an enhanced IBA1 staining in those animals ICV‐injected with NA (Figure 1g,g′) compared to those injected with saline (Figure 1f,h). As occurred in the DG, in a detailed look (Figure 1f′–i′) microglia in NA‐treated mice (Figure 1g′) seems to be morphologically different compared to cells from other groups. Moreover, in both structures DG and PVN, microglial morphological changes in NA‐injected animals (Figure 1c′,g′) seem to be at least partially reverted after PLX5622 treatment (Figure 1e′,i′). Further morphological studies were performed to address this issue.

**FIGURE 2:**
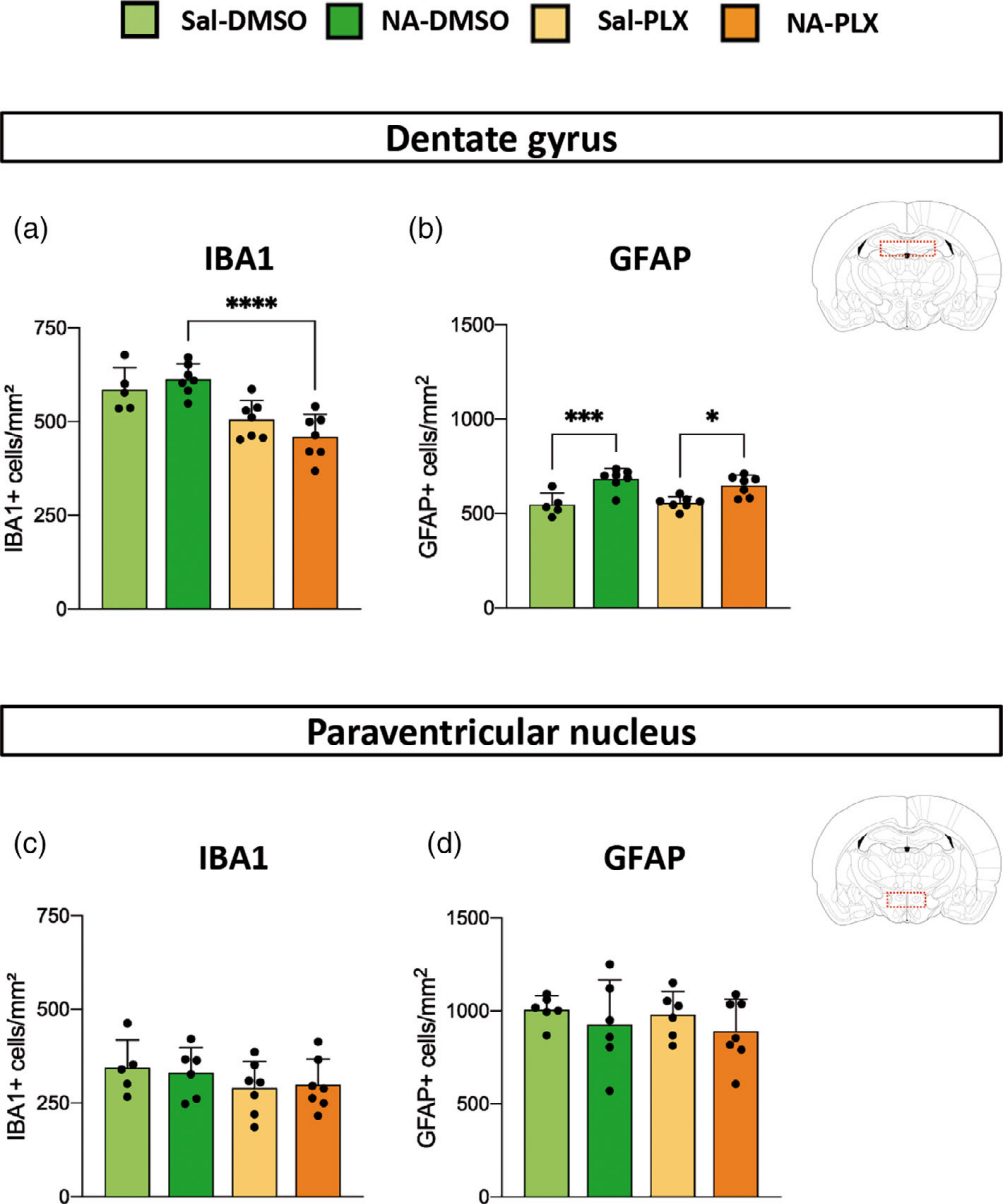
Microglia and astrocyte cell counts in the hippocampus and the hypothalamus after PLX5622 treatment and peripheral lipopolysaccharide (LPS) stimulation. (a–d) Cell counts were carried out in brain sections immunostained with IBA1 (a, c) for microglial counts, or GFAP (b, d) for astrocytes quantifications. Counts were performed in the dentate gyrus of the hippocampus (a, b) and the paraventricular nucleus (PVN) of the hypothalamus (c, d). Histograms show the mean ± SD of *n* = 5–7 mice per group. *p < .05; ***p < .001; ****p < .0001.

Cell density in the DG and the PVN was properly assessed by cell counts of IBA1‐stained microglia and GFAP‐stained astrocytes (Figure 2). In the DG microglia density was slightly reduced in those groups treated with PLX5622 (86% in Sal‐PLX and 75% in NA‐PLX, compared to Sal‐DMSO and NA‐DMSO respectively), reaching statistical significance only in NA‐injected mice (Figure 2a, NA‐DMSO vs. NA‐PLX). However, in the PVN microglia density was similar among experimental groups (Figure 2c). Microglial cell counts were also carried out in other brain regions (Figure S1a–d). In general, microglial density was slightly elevated (if any, depending on the brain region) in NA‐DMSO group, and reduced to basal levels after PLX5622 treatment. These results point out that peripheral LPS induces a mild microgliosis, which is enhanced by the previous NA‐induced inflammation. Besides, such enhanced microgliosis is prevented by low‐dose PLX5622 treatment.

To check the extent of microglial depletion provoked by PLX5622, one sentinel mouse from each experimental group was euthanized just upon PLX5622 treatment termination (Figure S2). Microglia population declined in all brain regions studied (septum, cortex, DG and periventricular hypothalamus), ranging from ≈50% in septum and hypothalamus to ≈40% in cortex and DG, thus confirming a partial depletion of microglia when using lower doses of PLX5622. Although microglial cell numbers recovered after PLX5622 withdrawal, in the DG the two groups treated with PLX5622 presented a slightly reduced number of microglia compared to their corresponding DMSO controls (Figure 2a). As mentioned above, we infer that PLX5622 prevented the LPS‐induced microgliosis, which was observed in various brain regions (see Figures 2a and S1a–d).

On the other hand, the population of astrocytes in PLX5622 treated mice remained unchanged, compared to the corresponding DMSO treated groups, both in DG (Figure 2b) and in PVN (Figure 2d), as well as in other brain regions (Figure S1e–h), which is in accordance with the selectivity of this compound for CSF1R and the specificity of CSF1R expression in myeloid cell lineage. Interestingly in hippocampal DG the ICV injection of NA provoked a slight but long‐lasting increase in the density of astrocytes, which endured irrespective of the PLX5622 treatment (Figure 2b), that could be probably attributed to sequelae of the severe neuroinflammation triggered by NA in tissues adjacent to the injection site.

Because the main goal of this work was to investigate a possible role of PLX5622 in reverting microglial priming, we further explored the microglial response to the second inflammatory challenge (LPS).

### 3.2 Morphological analysis of microglia suggests that low‐dose PLX5622 treatment reverts NA‐induced priming phenotype

**FIGURE 3:**
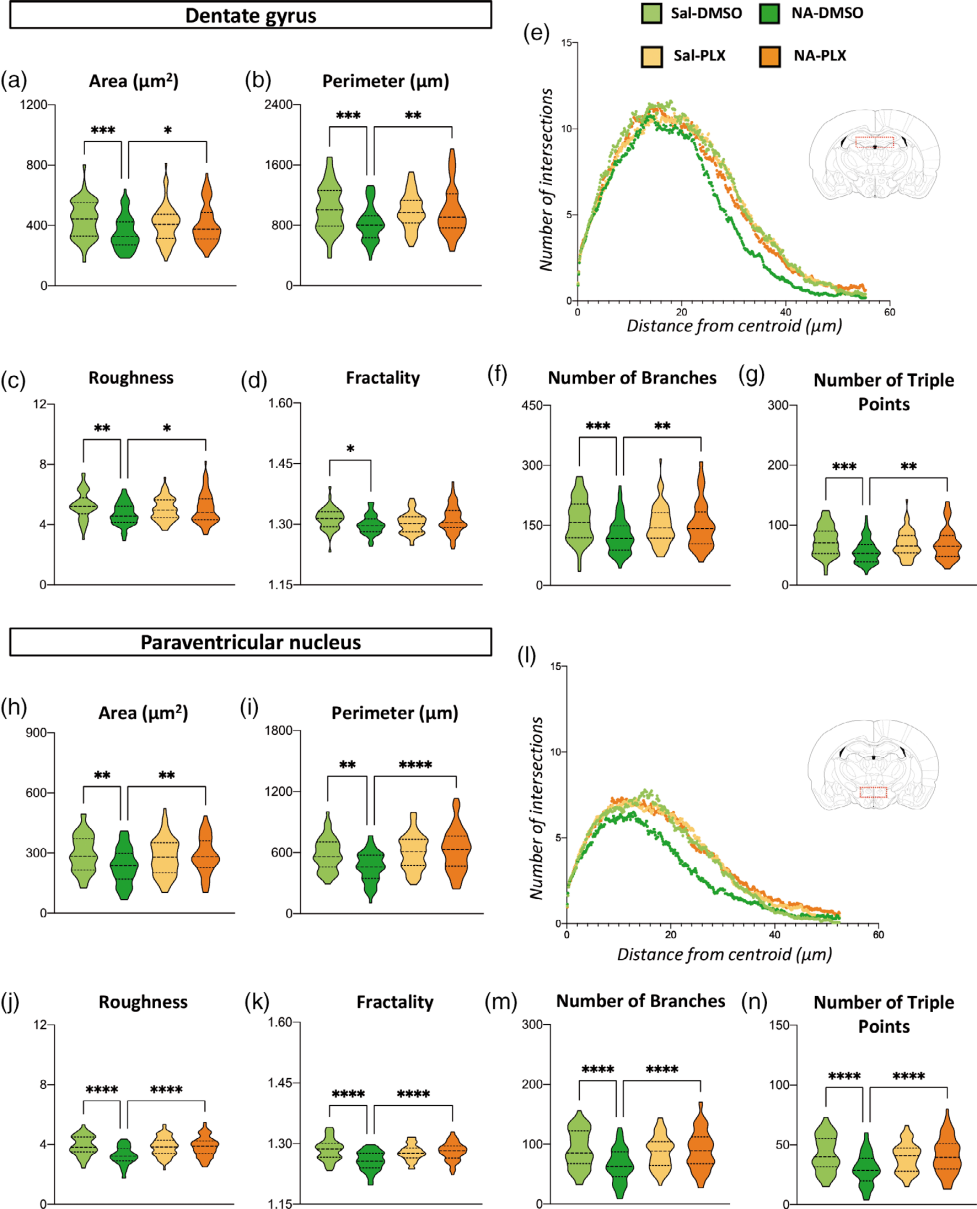
Morphological analysis of IBA1 immunostained microglial cells sampled from the hippocampal dentate gyrus (DG) and the paraventricular nucleus (PVN) of the hypothalamus. Morphological analysis was carried out by three different methods: (a–d, h–k) Fractal analysis, (e, l) Sholl analysis and (f,g, m,n) Skeleton analysis. Representative parameters are shown here; additional parameters are available as Supporting information. Data distribution of each parameter is presented as violin plot, which has been truncated at the maximum and minimum values of each dataset; the dashed line represents the median and the dotted line the quartiles. A total of *n* = 250–260 cells from DG and *n* = 240–250 cells from PVN, sampled from different animals within each experimental group, were analyzed. DMSO, dimethyl sulfoxide; neuraminidase; PLX, PLX5622; Sal, saline; NA. *p < .05; **p < .01; ***p < .001; ****p < .0001.

Morphology of microglia is a reliable indicator of its activation status (Fernandez‐Arjona et al., 2019). Individual microglial cells were sampled from IBA1‐stained histological sections to carry out an objective analysis of their morphology. Cells were sampled from the two areas of interest, the DG of the hippocampus and the PVN of the hypothalamus, and from different animals (*n* = 6–7) of each experimental group. Three different methods for morphological evaluation were employed, Fractal analysis, Sholl analysis and Skeleton analysis, resulting in a total of 27 different parameters. For simplicity, a selection of these parameters is shown here (Figure 3), but all of them are available as Supporting information (Figures S3 and S4).

First, 12 weeks after the initial ICV injection and 12 h after LPS stimulation, Fractal parameters *area*, *perimeter*, *roughness* and *fractality* decreased in NA‐injected mice compared to saline controls (Figure 3a–d,h–k). Interestingly, PLX5622 treatment of NA‐injected mice reversed the values of most of these parameters to those of microglia from saline‐injected animals, and this occurred both in DG and in PNV microglia (Figure 3a–d,h–k). Sholl analysis (Figure 3e,l) indicated shorter and fewer ramifications spanning from each cell centroid (indicated by a curve lower and shifted to the left in Figure 3e,l) in microglia from NA‐treated mice (dark green curves) compared to those of saline controls (light green curves). Microglia from NA‐injected mice treated with PLX5622 (orange curves) yielded curves similar to those of saline controls (yellow and light green curves). Finally, Skeleton parameters *number of branches* and *number of triple points* (Figure 3f,g,m,n) showed the same tendency, namely, (1) microglia from NA‐treated mice have less ramifications than saline controls and (2) PLX5622 treatment reverts such changes. In summary, morphological evaluation of microglia points out that NA‐injection provokes long‐lasting changes in microglia suggesting an enhanced activation upon LPS injection (i.e. primed state), while treatment with PLX5622 seems to restore microglia morphology to that found in control mice.

**FIGURE 4:**
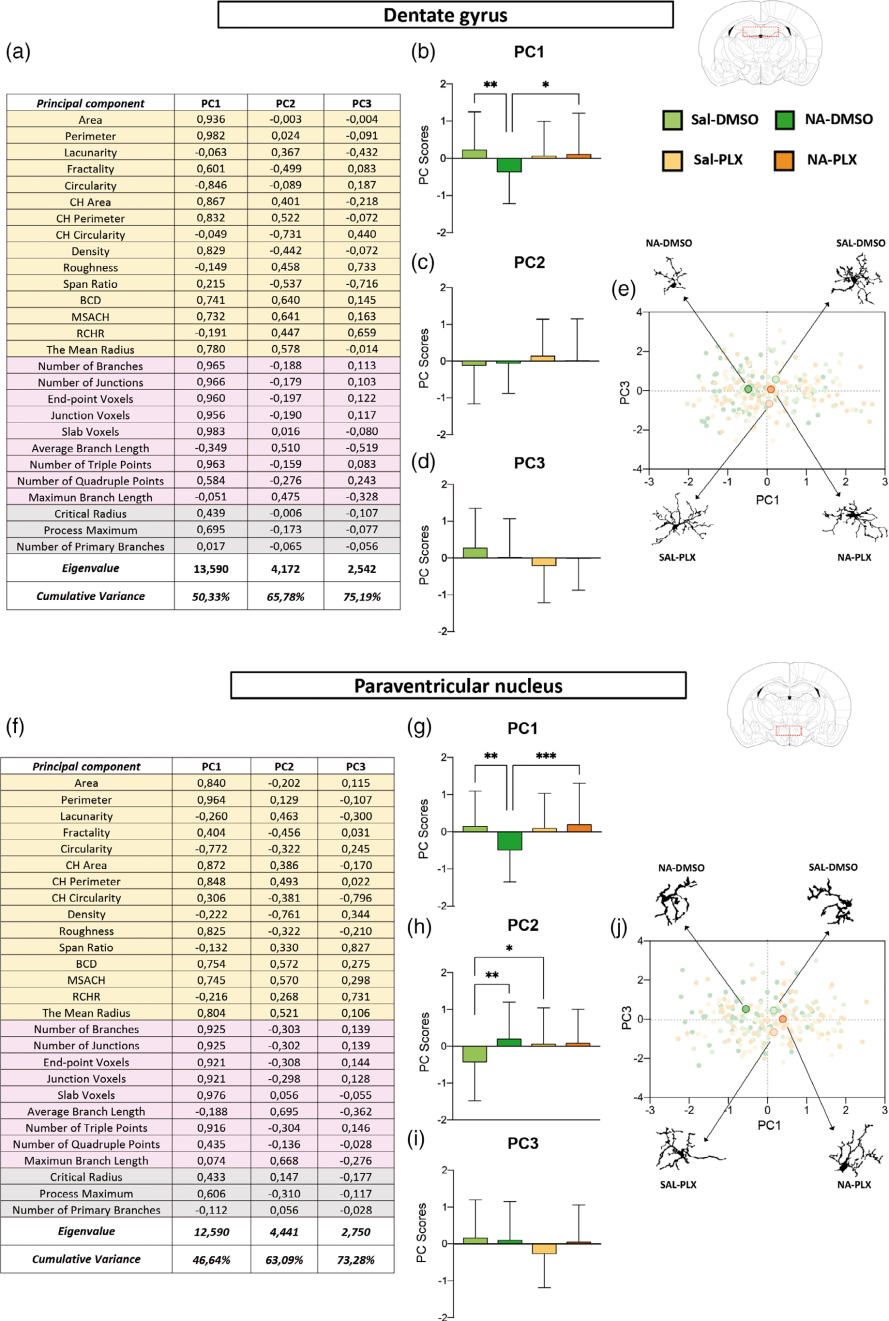
Principal components analysis (PCA) carried out with morphological parameters of microglial cells sampled from the dentate gyrus (DG) and the paraventricular nucleus (PVN) of the hypothalamus. (a, f) A total of 27 parameters were used for PCA, which were obtained from Fractal (shaded in yellow), Skeleton (shaded in pink), and Sholl (shaded in gray) morphological analysis. For the data obtained from DG microglia KMO = 0.849, *χ* ^2^ = 16349.691, and *p* < .0001. For the data from PVN microglia KMO = 0.832, *χ* ^2^ = 15200.137, and *p* < .0001. Tables present the PC scores for each parameter and the principal components (Eigenvalue >1) disclosed from the analysis, which have been named according to the highest parameters' scores for each component: PC1, PC2 and PC3. (b–d, g–i) Representation with histograms of the PC scores for PC1, PC2 and PC3 obtained from microglia sampled from dentate gyrus (b–d) and paraventricular nucleus (g–i). The bars represent the mean ± SD of *n* = 45–65 cells per experimental group. In order to reveal any significant difference between groups, a one‐way ANOVA and Tuckey post‐hoc test were conducted. (e, j) Distribution on the principal components plane of PC1 scores versus PC3 scores of *n* = 231 (sampled from DG) and *n* = 223 (sampled from PVN) individual microglial cells from the different experimental groups. The highlighted dots are the mean PC1–PC3 for each group. The filled profile of a representative cell from each group is depicted. DMSO, dimethyl sulfoxide; NA, neuraminidase; PLX, PLX5622; Sal, saline. *p < .05; **p < .01; ***p < .001.

For a more comprehensive evaluation of microglial morphology, a PCA was carried out with all 27 parameters, obtained by the above‐mentioned methods, in both microglia sampled from DG (Figure 4a) and from PVN (Figure 4f). PCA uncovered three components accounting for a total of 75.19% and 73.28% of variance for DG microglia and PVN microglia, respectively. Principal component 1 (PC1) was noticeably clarifying: strong positive correlations (high positive scores close to 1) were found in numerous parameters related to the cell size (*area, perimeter, convex hull area, convex hull perimeter*) and the complexity of ramifications (*number of branches, number of junctions, end‐point voxels, number of triple points*, etc.) In the case of PC2, the most relevant parameters (higher scores) where not so much related to the cell size or the complexity of ramifications but to its length (*bounding circle diameter*, *maximum span across the convex hull* (*MSCH*), along with *average branch length* and *maximum branch length*). PC3 was less conclusive, but according to the parameters scores (*span ratio*, *the ratio convex hull radii*) it seemed related to an elongated shape of the cell.

Besides, the mean values of the PC scores of all sampled cells were calculated and plotted in histograms, and comparisons between experimental groups were carried out by ANOVA (Figure 4b–d,g–i). Regarding PC1, microglia from NA‐injected mice were significantly different from the rest of groups, including microglia from NA‐injected and PLX5622 treated mice, both in DG (Figure 4b) and in PVN (Figure 4g) sampled microglia. This further confirms that (1) NA‐microglia is morphologically different from saline‐microglia, and (2) PLX5622 treatment reverts such morphology to that proper of saline controls.

In the case of PC2, no differences between groups were found in microglia from DG (Figure 4c). However, PVN microglia from NA‐treated mice was different from that of saline‐injected controls (Figure 4h), probably because of the shorter branch length of NA‐primed microglia compared to surveillant microglia present in saline‐injected mice. Also, PVN microglia in Sal‐DMSO group were found to be morphologically different from Sal‐PLX group regarding PC2, a result that might indicate some effect of PLX5622 treatment also on surveillant microglia, for example, a diminished sensibility to peripheral immune activation. Finally, the third component PC3 did not highlight microglial differences between any group (Figure 4d,i).

Also, the PC1 and PC3 scores of individual microglial cells were plotted on the principal components plane (Figure 4e,j). The mean PC1 and PC3 values in each experimental group, which are shown highlighted, allow to appreciate how PC1 discriminates microglia from NA‐DMSO group from microglia from the other three groups (Figure 4e,j), while PC3 does not allow such separation so clearly. A representative cell from each group is depicted in the periphery of the PC plane (Figure 4e,j). In DG, microglia from NA‐DMSO group are smaller and less ramified than microglia from saline‐injected mice (Sal‐DMSO and Sal‐PLX). More interestingly, NA‐PLX microglia are morphologically more similar to those of saline‐treated groups (Figure 4e). Profiles of cells sampled from PVN point in the same direction (Figure 4j), even though the general morphology of microglia in these two brain regions is clearly different.

Because morphological features of microglia are quite different depending on the brain region, cells were sampled from very specific locations, the DG and the PVN. Microglial morphology in these two structures was clearly different (Figure 1, compare b‐e with f‐i). Various parameters indicate that, compared to those cells in DG, cells in PVN are smaller (lower *area* and *perimeter*) and are less ramified (lower *number of branches*, *number of triple points* and *roughness*; Figure 3). Still, the results obtained in both regions point to the same conclusion: that NA‐induced neuroinflammation provokes changes in hippocampal and hypothalamic microglia consistent with a primed state, which can be reverted by the moderate PLX5622 treatment used here.

### 3.3 Expression of pro‐inflammatory genes in the hippocampus and the periventricular hypothalamus indicate that low‐dose PLX5622 treatment lessens inflammatory response to a secondary LPS peripheral stimulus

Twelve weeks after the administration of NA as priming stimulus, and 7 weeks after completion of PLX5622 treatment, a second stimulus was applied (peripheral LPS) and, 12 h later, the extent of the central inflammatory response was evaluated. The hippocampus and the periventricular hypothalamus were dissected out to obtain RNA extracts for gene expression quantification by qPCR, and protein extracts to be used for multiplex ELISA quantification of cytokines.

**FIGURE 5:**
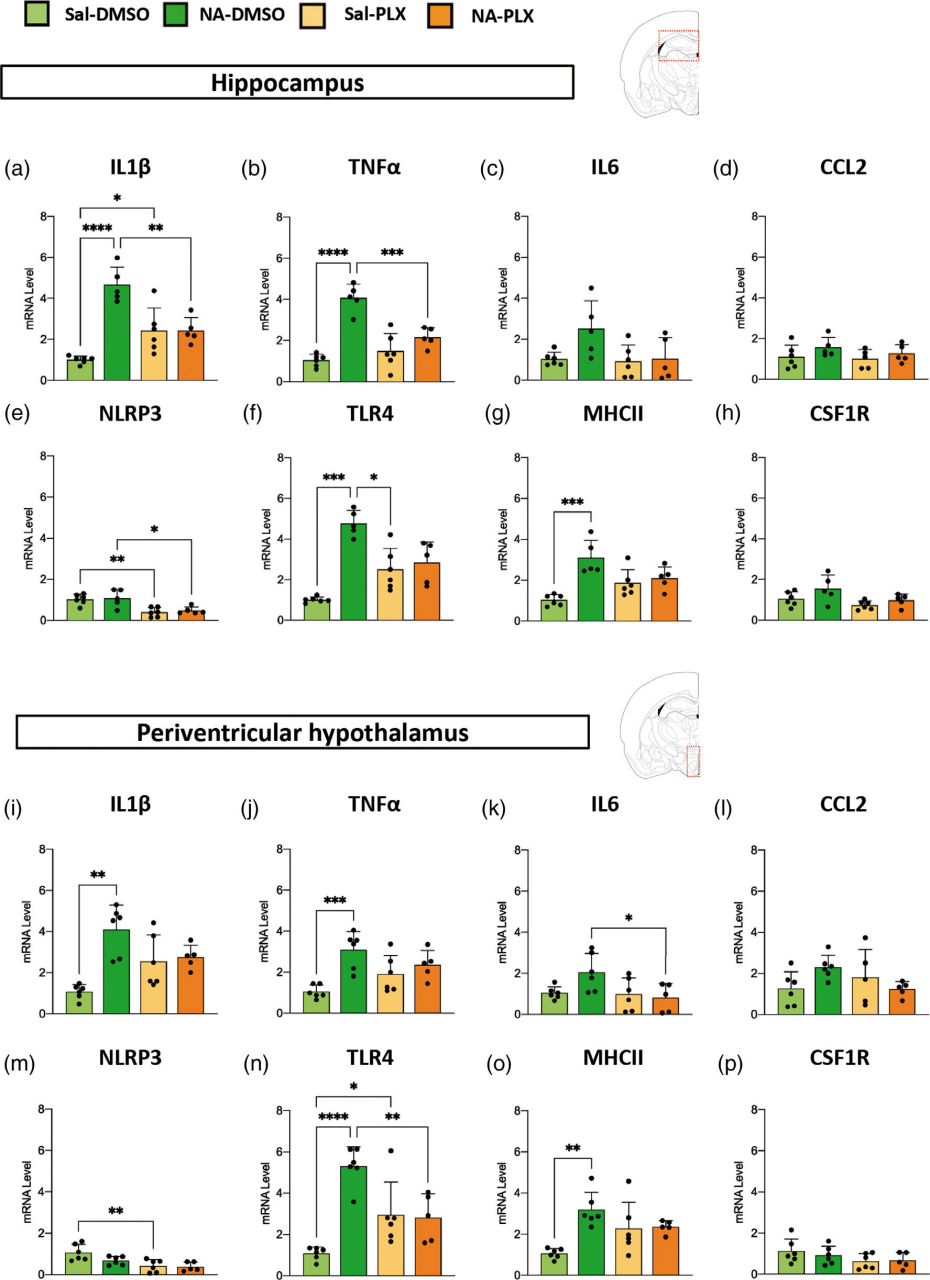
Gene expression in the hippocampus and the periventricular hypothalamus after PLX5622 treatment and peripheral lipopolysaccharide (LPS) stimulation. Mice were NA/saline‐injected and, 3 weeks later, treated with PLX5622 or vehicle DMSO. Seven weeks after treatment termination, LPS was injected intraperitoneal (IP) and mice euthanized 12 h later for tissue sampling. The mRNA levels of inflammation‐related genes and of the receptor target of PLX5622 (CSF1R) were quantified by quantitative polymerase chain reaction (qPCR), and expressed relative to the expression of the housekeeping gene GAPDH. (a–h) Quantification performed in hippocampus tissue. (i–p) Quantification performed in periventricular hypothalamic tissue. The histograms show the mean ± SD of *n* = 5–6 animals. DMSO, dimethyl sulfoxide; NA, neuraminidase; PLX, PLX5622; Sal, saline. *p < .05; **p < .01; ***p < .001; ****p < .0001.

Gene expression of cytokines IL1β, TNFα, IL6 and chemokine CCL2 were quantified by qPCR (Figure 5a–d,i–l). IL1β and TNFα expression were significantly increased in NA‐injected mice compared to saline‐injected controls, both in the hippocampus (Figure 5a,b) and the hypothalamus (Figure 5i,j). IL6 showed a similar tendency, but was not statistically significant (Figure 5c,k). Interestingly, PLX5622 treatment was able to reverse such increase at least partially, with statistically significant differences found between NA‐DMSO and NA‐PLX groups in the case of some genes/regions (IL1β/hippocampus, TNFα/hippocampus, IL6/hypothalamus; Figure 5a,b,k) but not others (IL6/hippocampus, IL1β/hypothalamus, TNFα/hypothalamus; Figure 5c,i,j). No differences were found in the expression of the chemokine CCL2 (Figure 5d,l).

Other pro‐inflammatory genes were evaluated as well, such as the inflammasome NLRP3, the pattern receptor TLR4 and the histocompatibility complex MHC II, a membrane protein complex considered a marker of microglial priming (Muccigrosso et al., 2016; Norden et al., 2015). The expression of NLRP3 was not increased by NA‐injection; however, it was decreased by the treatment with PLX5622 in the hippocampus of both saline‐ and NA‐injected mice (Figure 5e); a similar tendency was observed in the hypothalamus (Figure 5m). The expression of TLR4 (Figure 5f,n) and MHCII (Figure 5g,o) was significantly increased by NA‐induced priming, an increase that, in the case of TLR4, was reversed by treatment with PLX5622 (Figure 5f,n).

**FIGURE 6:**
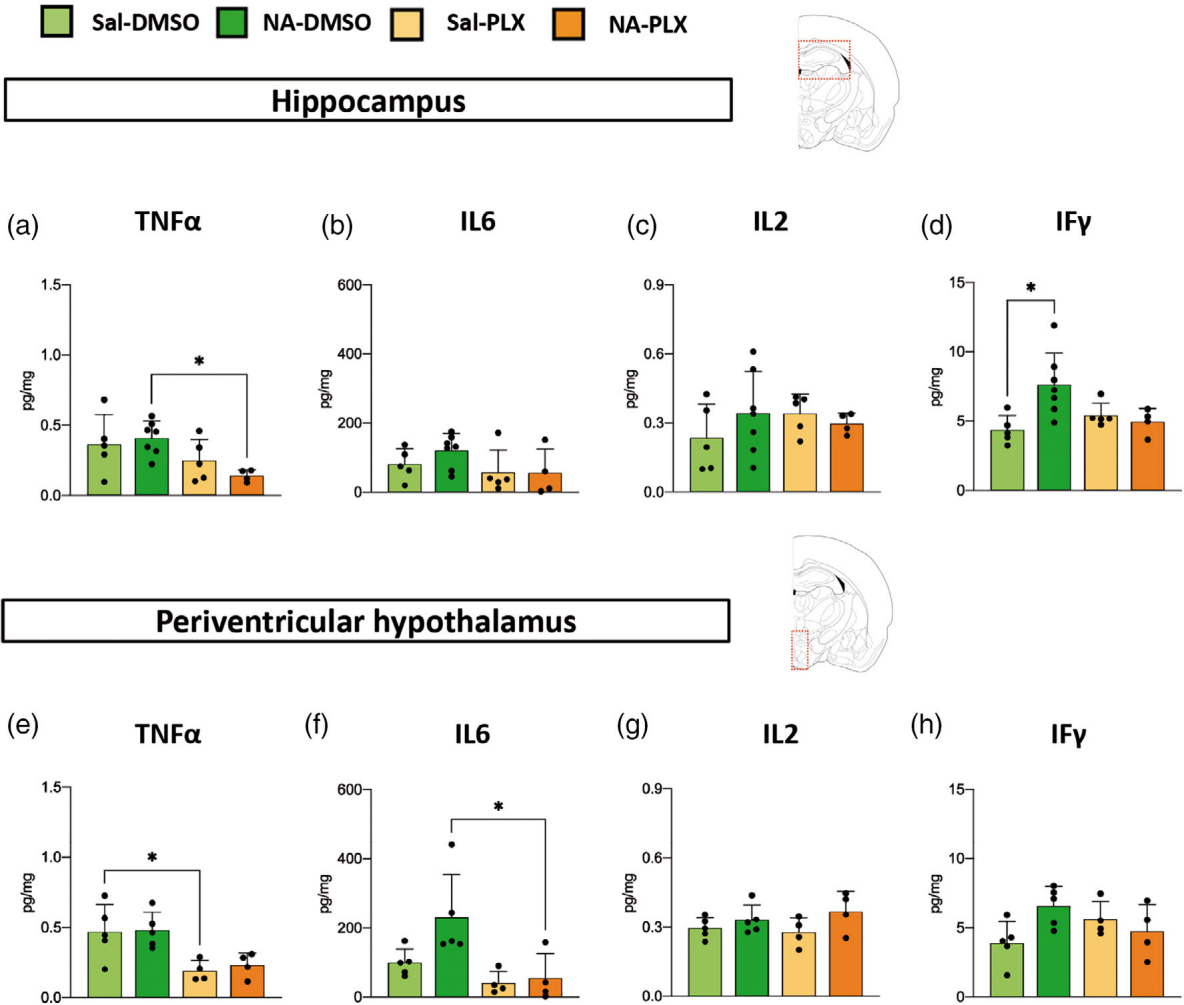
Quantification of cytokines in protein extracts from the hippocampus and the periventricular hypothalamus after PLX5622 treatment and peripheral lipopolysaccharide (LPS) stimulation. Mice were NA/saline‐injected and, 3 weeks later, treated with PLX5622 or vehicle DMSO. Seven weeks after treatment termination, LPS was injected intraperitoneal (IP) and mice euthanized 12 h later for tissue sampling. Cytokines were quantified from protein extracts by multiplex ELISA, and their amount expressed relative to the protein content of the extract. (a–d) Quantification performed in hippocampus tissue. (e–h) Quantification performed in periventricular hypothalamic tissue. The histograms show the mean ± SD of *n* = 5–7 mice. DMSO, dimethyl sulfoxide; NA, neuraminidase; PLX, PLX5622; Sal, saline. *p < .05.

In an attempt to have a more complete picture of the central inflammatory response to peripheral LPS stimulation, several cytokines were also quantified by multiplex ELISA, specifically TNFα, IL6, IL2 and IFγ (Figure 6). Although with moderate or not statistically significant differences, TNFα and IL6 presented a tendency to be decreased in PLX5622 treated groups, irrespective of previous NA‐induced inflammation (Figure 6a,b,e,f). No differences between groups were found in the protein levels of IL2 (Figure 6c,g) and IFγ (Figure 6d,h), apart from a slight induction of IFγ in the hippocampus of NA‐injected mice (Figure 6d).

Finally, gene expression of the receptor CSF1R, which is the target of PLX5622 compound, was also measured by qPCR. No differences were found between groups. However, a tendency (although not significant) to be decreased in PLX5622 treated mice was observed (Figure 5h,p).

To summarize, these results show that the expression of several pro‐inflammatory genes/proteins (IL1β, TNFα, TLR4, MHC II) was enhanced after peripheral LPS as a consequence of NA‐induced priming. Interestingly, transient treatment with a low dose of PLX5622 was able to prevent such increase (IL1β, TNFα, TLR4). Irrespective of the initial neuroinflammation, PLX5622 treatment provoked a moderate decline in LPS‐stimulated expression of various pro‐inflammatory genes/proteins, which was statistically significant only in some cases (NLRP3 in Figure 5e; TNFα in Figure 6a,e), while showed only a tendency in others (IL6 in Figure 6b,f). These effects were very similar in the two brain regions examined, the hippocampus and the periventricular hypothalamus. Overall, these results point out that PLX5622 employed at reduced doses seems to alleviate the LPS‐induced inflammatory response, and specifically the enhanced response of primed microglia, even as long as 7 weeks after completion of the treatment.

Finally, although it was not the scope of this work, the peripheral inflammatory response to the LPS challenge was assessed by measuring plasma levels of the cytokine IL1β. No differences were found between any experimental group (Figure S5), suggesting that the peripheral immune response was not altered by the low‐dose PLX5622 treatment used here.

## 4 DISCUSSION

Primed microglia have been proposed to be responsible for neurotoxic inflammatory responses that may drive a wide range of neurological alterations. Here we explored a strategy aimed to tame primed microglia, consisting in a treatment with the CSF1R inhibitor PLX5622 used at low doses. Higher doses of this drug have been previously used with the purpose of depleting the brain of microglia, mostly with the intention of assessing the contribution of these cells to physiological processes or to pathological scenarios (Dagher et al., 2015; Elmore et al., 2014; Huang et al., 2018; Spangenberg et al., 2019; Zhan et al., 2019). After PLX5622 provoked depletion of microglia, treatment interruption allows for microglia to repopulate the brain and, quite remarkably, the new cells seem to present a naïve profile, that is, they are more similar to unchallenged microglia devoid of immune memory (Elmore et al., 2014; Najafi et al., 2018; Zhan et al., 2019). Inspired by the work of some authors who used lower doses of PLX5622 that yielded a partial reduction of microglial population (Dagher et al., 2015; Feng et al., 2016; Mein et al., 2023; Najafi et al., 2018; Nissen et al., 2018), here we used a low dose of PLX5622 with the specific aim of reverting microglial priming back to a naïve state. Therefore, mice were first challenged by a priming stimulus consisting in the ICV injection of NA (Fernandez‐Arjona et al., 2022a; Granados‐Durán et al., 2015) which triggers acute and severe neuroinflammation, a process that was allowed to proceed until completely solved (≈3 weeks) before starting the treatment with PLX5622 (dose of 60 mg/kg administered IP during 12 days). Then, 7 weeks after PLX5622 withdrawal (period aimed to assess long‐term phenotype reversal, and long enough to allow for complete microglia repopulation) mice were again challenged (IP dose of LPS) to address the extent of the neuroinflammatory response. Our results demonstrate that (1) as expected, microglial cells primed by the NA challenge exhibited an enhanced response to peripheral LPS, compared to microglia from saline‐injected control mice, and (2) PLX5622 treatment was able to revert such enhanced microglial response to levels typical of microglia from control mice. Similar results were obtained both in the hippocampus and in the hypothalamus, the two brain regions selected for these studies. Microglial cell counts and morphological assessment (carried out in the hippocampal DG and in hypothalamic PVN) endorsed this conclusion, along with gene expression of inflammation‐related genes and, to a lesser extent, quantification of cytokines in brain tissue extracts. Although conclusions can be drawn from the experimental groups used, all of which were challenged with LPS, we are aware that the inclusion of mice not treated with LPS would have provided a more complete picture, with information on the consequences of PLX5622 treatment in a basal, unstimulated situation. These non‐challenged mice groups (a total of four) were omitted in order to reduce the number of animals used.

Morphological analysis of microglia has proved to be a very sensitive tool to evaluate the morphological changes that these cells undergo upon activation (Fernandez‐Arjona et al., 2017; Fernandez‐Arjona et al., 2019; Fernandez‐Arjona et al., 2022a; van Weering et al., 2023). Most morphological parameters obtained, as well as the PCA analysis carried out, indicated that after the LPS challenge microglia from NA‐DMSO mice were smaller and less ramified, a shape that is compatible with a more activated state, compared to microglia from NA‐injected and PLX‐treated mice or from saline‐injected controls. In fact, parameters related to the extent and complexity of ramifications were highly clarifying in the PC1 component, along with those related to the cell size (Figure 4a,f). Furthermore, the PC1 component scores allowed to discriminate NA‐DMSO microglia from the other experimental groups (Figure 4b,g). These results point out that treatment with low dose PLX5622 is able to revert NA‐primed microglia phenotype to a naïve state.

The central inflammatory response to the peripheral LPS challenge was further examined by the expression of cytokines in the hippocampus and the hypothalamus (specifically in the periventricular parenchyma of the III ventricle). Although statistical significance was not achieved in all cases (particularly in cytokine protein quantifications, Figure 6), the results showed that the IL1β, TNFα and IL6 responses were enhanced in NA‐DMSO mice compared to NA‐PLX and saline control mice, thus confirming the effect of PLX5622 in reverting the primed state of microglia. The expression of other genes related to neuroinflammation (NLRP3, TLR4) and microglial priming (MHCII) pointed to the same conclusion. Gene expression, however, retrieved intriguing results: some induction of IL1β (Figure 5a,i) and TLR4 (Figure 5f,n) in saline controls treated with PLX5622, compared to mice not treated with PLX5622. These results might suggest that PLX5622 could induce a mild inflammatory sensitization/activation of naïve microglia. The expression of the inflammasome NLRP3 (Figure 5e,m), conversely, pointed out that PLX5622 lessens the pro‐inflammatory response after the LPS challenge in all cases. In spite of the intriguing behavior of particular genes, overall our gene/protein expression results demonstrate that PLX5622 treatment is able to prevent the enhanced response of NA‐primed microglia to the peripheral LPS challenge, which is in line with the microglial morphological results discussed above. As the expression of the receptor CSF1R is restricted to cells of the myeloid lineage (Stanley & Chitu, 2014), in the brain PLX5622 is well known to specifically target microglia, along with other less abundant brain macrophages located in the perivascular space, meninges and choroid plexus (Spiteri et al., 2022; Van Hove et al., 2019). Therefore, we can infer that the suppression of the enhanced inflammatory response to LPS is related to PLX5622 actions on brain‐residing myeloid cells, in particular the most abundant microglia. In fact, astrocyte cell counts carried out in hippocampal DG and hypothalamic PVN showed unaltered astrocyte density. However, although PLX5622 does not directly impact astrocytes, and because microglia and astrocytes are subject to extensive communication and interaction, any effect on microglia could also affect astrocytes. Thus, PLX5622 actions on microglia might affect also astrocytes responses to LPS, therefore, we cannot rule out that astrocytes may be contributing as well to the observed changes in gene/protein expression. Further specific evaluation of these two cell populations (such as the morphological analysis carried out on microglial cells) would help to clarify this point.

CSF1R inhibitors have emerged as useful tools to study the role of microglia in various pathologies. For such end, most experimental paradigms aim to evaluate a particular function in the absence of microglia, using inhibitor dosage high enough to achieve a significant degree of microglial depletion. Therefore, many experiments using PLX compounds are carried out under a situation of microglial depletion, thus highlighting microglial function both in the normal brain and in pathological scenarios (Asai et al., 2015; Basilico et al., 2022; Fu et al., 2020; Janova et al., 2018; Qu et al., 2017; Sariol et al., 2020; Sawicki et al., 2019; Spangenberg et al., 2019; Szalay et al., 2016; Tan et al., 2022; Wheeler et al., 2018; Witcher et al., 2021). Experimental animals treated with PLX5622 suffer a considerable reduction in the population of microglial cells, which may reach values of 90%–99% when using large doses of PLX5622 applied for long periods of time (Huang et al., 2018; Spangenberg et al., 2019; Zhan et al., 2019). Typically, a PLX5622 treatment aimed to microglia depletion consists of 1200 mg/kg of chow, during 7–14 days, which results in an almost complete elimination of microglia (Basilico et al., 2022; Dagher et al., 2015; Rice et al., 2017). Discordances in the percentage of depletion reported by different authors could be ascribed to differences in the dose/length of the treatment; however, the manufacturer and/or batch of PLX5622 needs to be considered as well (Karaahmet et al., 2022). The mild treatment of PLX5622 used here (60 mg/kg body weight, administered IP during 12 days) provoked, as intended, a microglial depletion ranging between 40% and 50% (depending on the brain region studied), in line with reports by other authors that used low doses (300 mg/kg of chow) of PLX5622 (Dagher et al., 2015; Feng et al., 2016; Mein et al., 2023; Nissen et al., 2018). Although PLX5622 is usually administered in chow, we chose the IP administration route (Vukojicic et al., 2019) to ensure that all animals consistently received the dose selected (60 mg/kg body weight) as animals may not eat the same amount of chow. Considering the average amount of chow (≈4 g chow) eaten by a mouse (≈30 g bw) each day, a PLX5622 diet containing 300 mg/kg would correspond to a dose of ≈40 mg/kg bw, which is similar to that used in this work. Thus, moderate doses of PLX5622 (40–60 mg/kg bw) administered either by diet, IP or by oral gavage (Campos et al., 2022; Lee et al., 2018) prevent the massive depletion of microglia provoked by higher doses of this compound. Although complete depletion of microglia is a powerful tool to investigate microglia functions, it also represents a compromised situation that challenges immune responses to infectious agents within the brain (Fekete et al., 2018; Funk & Klein, 2019; Mangale et al., 2020; Sanchez et al., 2021; Seitz et al., 2018; Waltl et al., 2018; Wheeler et al., 2018).

On the contrary, we and other works pursued a temporary reduction of microglial population, benefiting from the transient nature of CSF1R inhibition. Therefore, we assessed the recovery of microglial population 7 weeks after PLX5622 treatment completion. As expected, microglial numbers in PLX5622‐treated mice were broadly similar to those of DMSO vehicle‐treated mice. In fact, it has been reported that upon PLX5622 (or PLX3397) withdrawal microglia starts proliferating almost immediately, reaching normal densities after 2–3 weeks (Elmore et al., 2014; Weber et al., 2019). Because we aimed to evaluate long‐term reversal of microglial priming, we allowed for a quite long (7 weeks) withdrawal period. Although the origin of these new microglia has been a matter of debate, it is widely accepted that they arise mostly from resident microglial cells surviving PLX5622 treatment (Elmore et al., 2014; Huang et al., 2018; Zhou et al., 2022); a contribution of peripheral monocytes/macrophages might as well occur (Lund et al., 2018; Najafi et al., 2018). Of note, we found a slightly higher microglial density in NA‐injected DMSO‐treated mice in various brain regions studied, which we interpret as a microgliosis response triggered by the peripheral LPS stimulation, a response which was enhanced in NA‐primed brains and, interestingly, suppressed by PLX5622 treatment. Moreover, a slightly reduced microglial density was observed in some brain regions (DG and amygdala) in both (NA and saline) PLX5622‐treated groups, suggesting that PLX5622 treatment might lessen LPS‐induced microglial response in all scenarios. In fact, the PC2 component that emerged in the PCA analysis of hypothalamic microglia showed a morphological difference between saline‐DMSO and saline‐PLX groups. It should be mentioned that a mild neuroinflammation (and therefore a potential risk of priming) occurs also in saline‐injected controls as a result of ICV surgery (Fernandez‐Arjona et al., 2022a; Leon‐Rodriguez et al., 2022), a situation that could benefit from PLX5622 actions. Alternatively, the possibility that complete repopulation might be, at least to some extent, region‐specific should be considered.

Besides provoking a turnover in microglial population, CSF1R inhibition modifies the phenotype of microglia once PLX5622 treatment is concluded. Evaluation of microglial phenotype after transient PLX5622 treatment suggests a switch to a homeostatic profile (Elmore et al., 2015, Elmore et al., 2018; Rice et al., 2017). Likewise, our results point out that PLX5622 treatment was able to revert the primed phenotype of microglia, which had been previously induced by acute severe neuroinflammation. Besides, this was achieved with the significant advantage of using low doses of PLX5622. Moreover, such phenotype reversal was sustained, as we evaluated the response to an LPS challenge up to 7 weeks after PLX5622 treatment termination; in this sense, some works report phenotype reversal as far as after 4–5 weeks from treatment withdrawal (Elmore et al., 2018; Henry et al., 2020; Mein et al., 2023).

The benefits of microglial phenotypic switch associated with depletion‐repopulation have been explored in various animal models. Microglia turnover restored synaptic density and rescued cognitive deficits in Alzheimer's disease models (Dagher et al., 2015; Wang et al., 2023); some authors, however, did not observe these improvements (Karaahmet et al., 2022). Also, repopulating microglia exerted a positive modulation of the brain milieu after traumatic brain injury, reducing the inflammatory status of microglia, facilitating brain repair and improving motor, behavioral and cognitive recovery (Bray et al., 2022; Henry et al., 2020; Willis et al., 2020). A similar positive outcome was reported as well in a model of stroke (Szalay et al., 2016) and in a model of injury‐triggered neuropathic pain (Lee et al., 2018). Moreover, behavioral sensitization (or priming) induced by cocaine was reversed by pharmacological microglial turnover (da Silva et al., 2021), which raises the possibility of using CSF1R inhibition to alleviate the consequences that drug abuse imposes on the brain. Interestingly, although some authors report functional improvement and phenotypic reversal in age‐induced microglial priming (Elmore et al., 2018), other works find a resistance to a sustained phenotypic switch in the aged brain, highlighting the importance of the nervous system milieu and its impact on microglial inflammatory phenotype (O'Neil et al., 2018). Thus, as has been previously pointed (O'Neil et al., 2018), the benefits from phenotypic modulation after microglia turnover provoked by CSF1R inhibition are highly dependent on the context, with notable differences reported depending on age, chronicity of the disease, or the type of stimulus that triggered neuroinflammation and priming (pathogen, psychologic stressor, injury, etc.).

An interesting aspect of our results is that primed phenotype reversal is feasible without the need of fully depleting the brain of microglia, which represents a hazardous situation for experimental animals and would be highly objectionable in a clinical setting. In fact, treatment of experimental animals with other types of CSF1R inhibitors (e.g. GW2580 or sCSF1Rinh) which do not affect microglial survival (and therefore do not provoke depletion) but halt their proliferation, promotes an anti‐inflammatory phenotype, reduces microgliosis and macrophage infiltration, improves recovery from spinal cord injury (Gerber et al., 2018), and slows the progression of amyotrophic lateral sclerosis (Martínez‐Muriana et al., 2016) or multiple sclerosis (Hagan et al., 2020), thus highlighting the potential therapeutic use of CSF1R inhibitors for human diseases. Thus, these works suggest that CSF1R inhibition promotes an immunomodulatory phenotype in microglial cells, which is independent of the population turnover that such inhibition may provoke. In this sense, our results support the interest of further investigating even lower doses of PLX5622 aimed at modulating the pro‐inflammatory activation of microglia with a minimal impact on cell density. Additionally, lower doses and/or limited treatments would most probably reduce, or even prevent, the off‐target impact of CSF1R inhibition on peripheral immune cells, a frequently mentioned drawback of such inhibitors (Lei et al., 2020; Okojie et al., 2023). Interestingly, in some scenarios limited CSF1R inhibition in peripheral cells may be as well beneficial for specific brain pathologies, as it diminishes infiltration of reactive monocytes/macrophages (Martínez‐Muriana et al., 2016; Spiteri et al., 2022). Although we did not sample immune peripheral cells at the moment of mice sacrifice, we did take plasma samples and measured the levels of IL1β (Figure S5). We did not observe differences in the IL1β response triggered by LPS in those animals treated with PLX5622 compared to the DMSO controls, which suggests that peripheral immune cells were not significantly affected by the treatment or, in case they were, they had recovered after the PLX5622 withdrawal period. A more detailed evaluation of peripheral immune cells would be required to confirm this end.

CSF1R inhibitors have attracted great interest as promising therapeutic agents, particularly in cancer, mostly because of their ability to modulate tumor‐associated macrophages, which typically present an immunosuppressive phenotype that aids tumor progression (Lv et al., 2024; Mok et al., 2014; Strachan et al., 2013; Xu et al., 2013). In fact, in 2019 the FDA approved PLX3397 (pexidartinib) for the treatment of tenosynovial giant cell tumors, and intense investigation in this field is ongoing (Cannarile et al., 2017; Wen et al., 2023). However, these studies did not report the impact of CSF1R inhibitor treatment on microglia in the human brain. A single clinical trial approached the use of PLX3397 in recurrent glioblastoma patients, a treatment that unfortunately showed no efficacy, in spite of the good penetrability of the compound to the brain. In this study microglia were examined in tumor surgical resection samples; histological results showed limited, if any, impact on microglia (Butowski et al., 2016).

While the results of this work provide evidence that PLX5622 used at a low dose can revert microglial priming, new stimulating questions arise. First, we observed that PLX5622 treatment seems to have some very subtle effect in microglia from saline‐injected animals, as shown by qPCR (Figure 5) and multiplex ELISA (Figure 6), which should be further scrutinized. How does PLX5622 impact on surviving microglia compared to regenerated microglia? Answering this question requires to be able to discriminate between both microglial populations. The phenotypic profile (including LPS responsiveness) and the gene expression signature of these two populations will help clarify how PLX5622 might modify microglia phenotype. Finally, the functional benefit of microglial priming reversal with low dose PLX5622 in disease/injury animal models should be of priority interest. Our results demonstrate that, after an acute inflammatory event that induces microglial priming, a moderate treatment (both in terms of dose and time) with PLX5622, which does not completely ablate microglia, is able to restore the homeostatic phenotype of primed microglia. Besides, this homeostatic profile is sustained over time, as it was evaluated up to 7 weeks after treatment completion. Exhaustive or chronic CSF1R inhibition compromises immune responses both central and peripherally. Conversely, dose and duration‐controlled inhibition preserves myeloid cell populations and promotes their immunomodulatory phenotype (Mein et al., 2023). Because of the limited availability of molecules able to cross the blood–brain barrier to target brain cells, these results support the previously suggested idea of reconsidering CSF1R inhibitors as therapeutic modulators of primed, hyperactive, or chronically activated microglia, which is a common feature in many disease conditions (reviewed in Han et al., 2022; Shi et al., 2022; Tarale & Alam, 2022; Weyer et al., 2024). A careful appraisal of the specific context concurring in each particular disease, considering the nature of the brain insult (infection, injury, stroke, neurodegeneration, etc.) and its persistence over time, and the impact of the treatment on both central and peripheral myeloid cells, should help to address the advisability of exploring the therapeutic potential of a moderate blockade of CSF1R signaling by using low doses of CSF1R inhibitors.

## AUTHOR CONTRIBUTIONS

María Dolores López‐Ávalos, Jesús M. Grondona: Conceptualization. Ana León‐Rodríguez, Jesús M. Grondona: Conducting experiment. Ana León‐Rodríguez, Sonia Marín‐Wong, Manuel F. López‐Aranda: Sample processing, quantifications and analysis. Ana León‐Rodríguez: Statistical analisis. María Dolores López‐Ávalos, Jesús M. Grondona: Supervision. María Dolores López‐Ávalos: Writing original draft. María Dolores López‐Ávalos, Jesús M. Grondona, Ana León‐Rodríguez, Sonia Marín‐Wong, Manuel F. López‐Aranda: Manuscript review and editing.

## CONFLICT OF INTEREST STATEMENT

The authors declare no conflict of interest.

## Supporting information


**Figure S1.** Microglia (a‐d) and astrocyte (e‐h) cell counts in several brain regions after PLX5622 treatment followed by 7 weeks of withdrawal. Animals received an IP injection of LPS 12 h prior to sacrifice. Histograms show the mean ± SD of *n* = 6–7 mice. Sal, saline; NA, neuraminidase; DMSO, dimethyl sulfoxide; PLX, PLX5622. *p < 0.05, **p < 0.01, ***p < 0.001, ****p < 0.0001.
**Figure S2.** IBA1‐positive microglia cell counts at the end of the 12‐day PLX5622 treatment. A single animal per experimental group was sacrificed to evaluate the extent of microglial depletion. Each bar is the mean of 4–8 sections counted. Microglial depletion occurred in all regions examined: (a) septum, (b) cortex (contralateral to the injected ventricle), (c) dentate gyrus and (d) periventricular hypothalamus. wPercentages on top of the bars indicate the estimated reduction in microglial cell number provoked by PLX5622 treatment, relative to the corresponding DMSO control. Sal, saline; NA, neuraminidase; DMSO, dimethyl sulfoxide; PLX, PLX5622. *p < 0.05, **p < 0.01, ***p < 0.001, ****p < 0.0001.
**Figure S3.** Morphological analysis of IBA1 stained microglial cells sampled from the paraventricular nucleus of the hypothalamus. Morphological analysis was carried out by three different methods: Fractal analysis (yellow area), Skeleton analysis (pink area) and Sholl analysis (gray area). Data distribution of each parameter is presented as violin plot, where the dashed line represents the median and the dotted line represents the quartiles. *N* = 240–250 cells were sampled from different animals within each experimental group. Sal, saline; NA, neuraminidase; DMSO, dimethyl sulfoxide; PLX, PLX5622. *p < 0.05, **p < 0.01, ***p < 0.001.
**Figure S4.** Morphological analysis of IBA1 stained microglial cells sampled from the dentate gyrus of hippocampus. Morphological analysis was carried out by three different methods: Fractal analysis (yellow area), Skeleton analysis (pink area) and Sholl analysis (gray area). Data distribution of each parameter is presented as violin plot, where the dashed line represents the median and the dotted line represents the quartiles. *N* = 240–250 cells were sampled from different animals within each experimental group. Sal, saline; NA, neuraminidase; DMSO, dimethyl sulfoxide; PLX, PLX5622. *p < 0.05, **p < 0.01, ***p < 0.001.
**Figure S5.** Serum IL1β in mice 12 h after an IP challenge with LPS. Three months before, mice were ICV injected with NA or saline, and later treated with PLX or vehicle. Histograms show the mean ± SD of *n* = 11–13 mice. Sal, saline; NA, neuraminidase; DMSO, dimethyl sulfoxide; PLX, PLX5622.

**Table S1.** Sequence of the primers used for qPCR.

## Data Availability

The data that support the findings of this study are available from the corresponding author upon reasonable request.
