# Supplementary material for: Long‐term reprogramming of primed microglia after moderate inhibition of CSF1R signaling
Source: Glia. 2024 Oct 24;73(1):175–95. doi: 10.1002/glia.24627 (PMC11660525; doi:10.1002/glia.24627)
Supplement: Supplementary file 1 — Figure S1. Microglia (a‐d) and astrocyte (e‐h) cell counts in several brain regions after PLX5622 treatment followed by 7 weeks of withdrawal. Animals received an IP injection of LPS 12 h prior to sacrifice. Histograms show the mean ± SD of n = 6–7 mice. Sal, saline; NA, neuraminidase; DMSO, dimethyl sulfoxide; PLX, PLX5622. *p < 0.05, **p < 0.01, ***p < 0.001, ****p < 0.0001. Figure S2. IBA1‐positive microglia cell counts at the end of the 12‐day PLX5622 treatment. A single animal per experimental group was sacrificed to evaluate the extent of microglial depletion. Each bar is the mean of 4–8 sections counted. Microglial depletion occurred in all regions examined: (a) septum, (b) cortex (contralateral to the injected ventricle), (c) dentate gyrus and (d) periventricular hypothalamus. wPercentages on top of the bars indicate the estimated reduction in microglial cell number provoked by PLX5622 treatment, relative to the corresponding DMSO control. Sal, saline; NA, neuraminidase; DMSO, dimethyl sulfoxide; PLX, PLX5622. *p < 0.05, **p < 0.01, ***p < 0.001, ****p < 0.0001. Figure S3. Morphological analysis of IBA1 stained microglial cells sampled from the paraventricular nucleus of the hypothalamus. Morphological analysis was carried out by three different methods: Fractal analysis (yellow area), Skeleton analysis (pink area) and Sholl analysis (gray area). Data distribution of each parameter is presented as violin plot, where the dashed line represents the median and the dotted line represents the quartiles. N = 240–250 cells were sampled from different animals within each experimental group. Sal, saline; NA, neuraminidase; DMSO, dimethyl sulfoxide; PLX, PLX5622. *p < 0.05, **p < 0.01, ***p < 0.001. Figure S4. Morphological analysis of IBA1 stained microglial cells sampled from the dentate gyrus of hippocampus. Morphological analysis was carried out by three different methods: Fractal analysis (yellow area), Skeleton analysis (pink area) and Sholl analysis (gray area) [file GLIA-73-175-s002.pptx]

## Slide 1
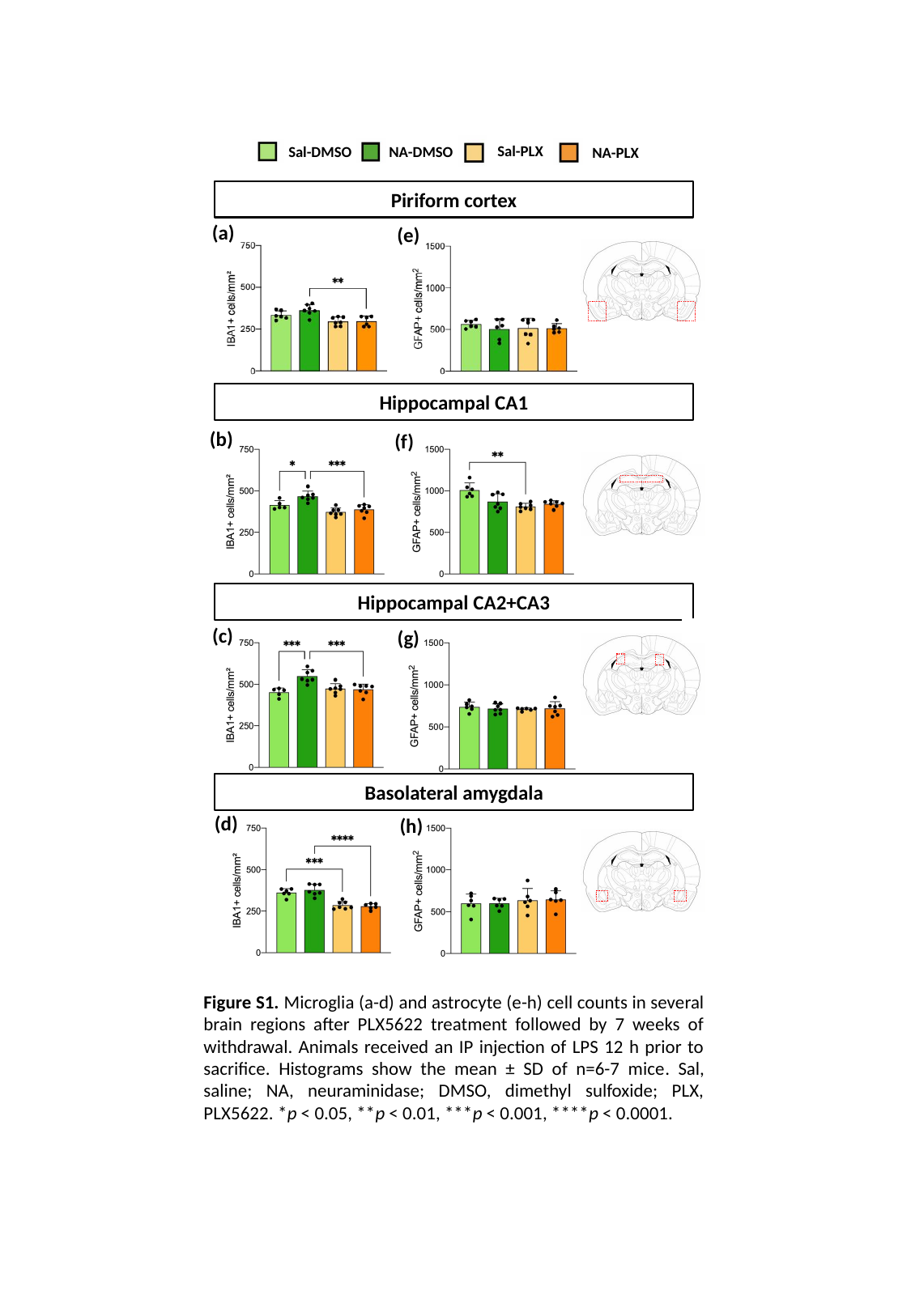

Sal-PLX
Sal-DMSO
NA-DMSO
NA-PLX
Piriform cortex
(a)
(e)
Hippocampal CA1
(b)
(f)
Hippocampal CA2+CA3
(c)
(g)
Basolateral amygdala
(d)
(h)
Figure S1. Microglia (a-d) and astrocyte (e-h) cell counts in several brain regions after PLX5622 treatment followed by 7 weeks of withdrawal. Animals received an IP injection of LPS 12 h prior to sacrifice. Histograms show the mean ± SD of n=6-7 mice. Sal, saline; NA, neuraminidase; DMSO, dimethyl sulfoxide; PLX, PLX5622. *p < 0.05, **p < 0.01, ***p < 0.001, ****p < 0.0001.

## Slide 2
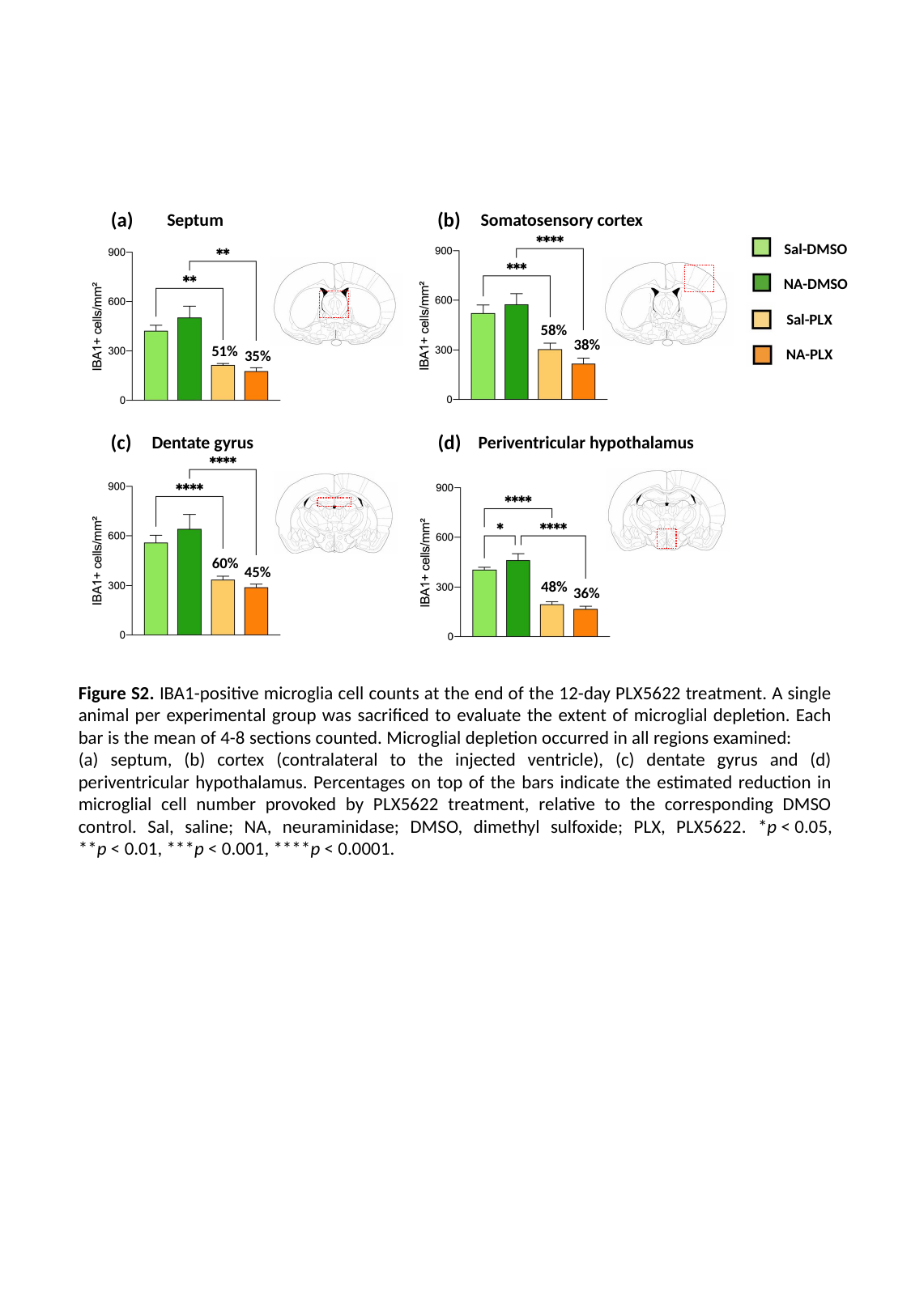

(a)
(b)
Septum
Somatosensory cortex
Sal-DMSO
NA-DMSO
Sal-PLX
NA-PLX
58%
38%
51%
35%
(c)
(d)
Dentate gyrus
Periventricular hypothalamus
60%
45%
48%
36%
Figure S2. IBA1-positive microglia cell counts at the end of the 12-day PLX5622 treatment. A single animal per experimental group was sacrificed to evaluate the extent of microglial depletion. Each bar is the mean of 4-8 sections counted. Microglial depletion occurred in all regions examined: (a) septum, (b) cortex (contralateral to the injected ventricle), (c) dentate gyrus and (d) periventricular hypothalamus. Percentages on top of the bars indicate the estimated reduction in microglial cell number provoked by PLX5622 treatment, relative to the corresponding DMSO control. Sal, saline; NA, neuraminidase; DMSO, dimethyl sulfoxide; PLX, PLX5622. *p < 0.05, **p < 0.01, ***p < 0.001, ****p < 0.0001.

## Slide 3
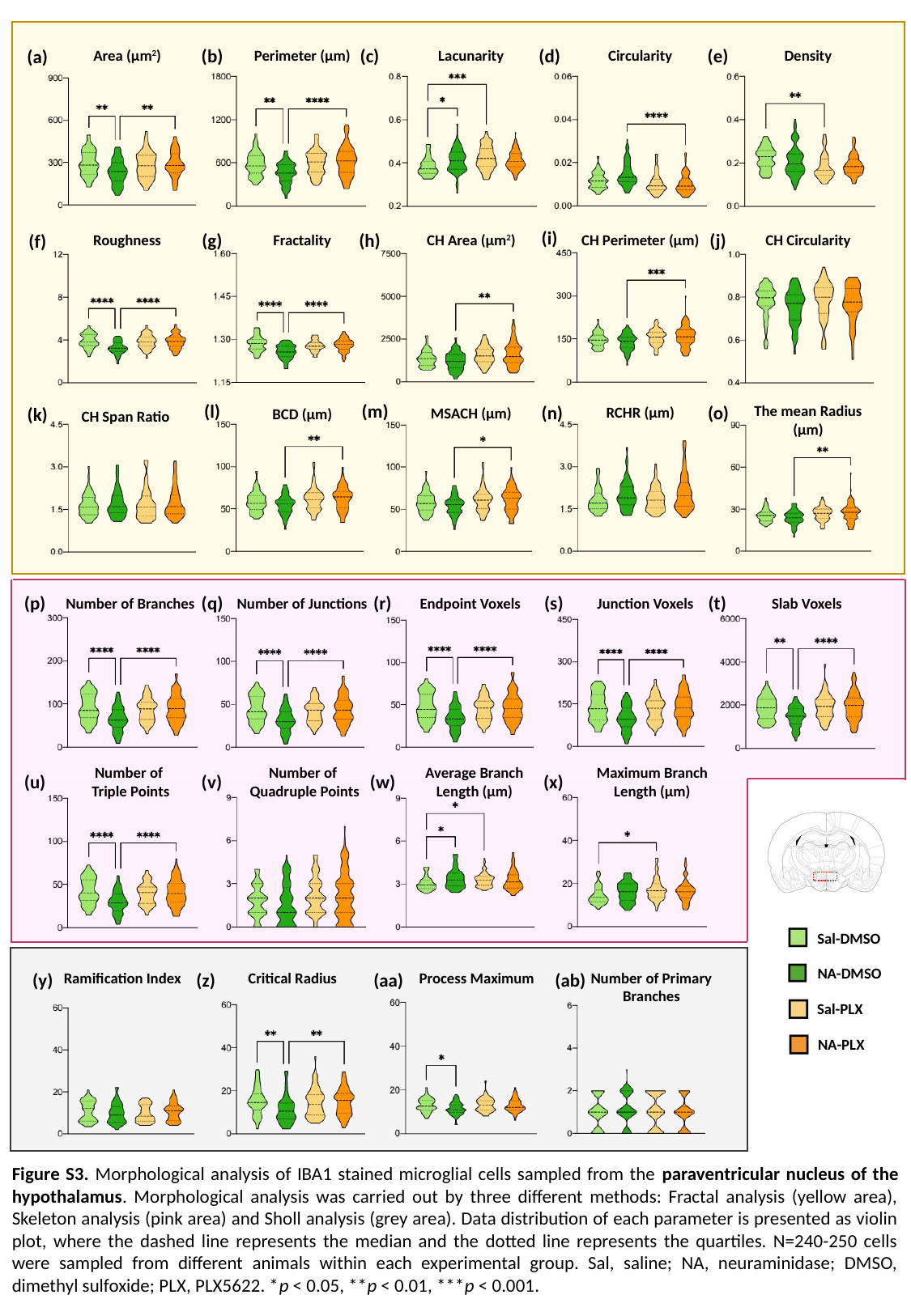

(b)
(c)
(d)
(e)
(a)
Area (μm2)
Perimeter (μm)
Lacunarity
Circularity
Density
(i)
(g)
(h)
(j)
(f)
Roughness
Fractality
CH Area (μm2)
CH Perimeter (μm)
CH Circularity
(m)
(l)
(n)
(o)
The mean Radius (μm)
RCHR (μm)
(k)
MSACH (μm)
BCD (μm)
CH Span Ratio
(p)
(q)
(r)
(s)
(t)
Number of Branches
Number of Junctions
Endpoint Voxels
Junction Voxels
Slab Voxels
Number of
Triple Points
Number of
Quadruple Points
Average Branch Length (μm)
Maximum Branch Length (μm)
(u)
(v)
(w)
(x)
Sal-DMSO
NA-DMSO
Sal-PLX
NA-PLX
(y)
Ramification Index
(z)
Critical Radius
(aa)
Process Maximum
(ab)
Number of Primary Branches
Figure S3. Morphological analysis of IBA1 stained microglial cells sampled from the paraventricular nucleus of the hypothalamus. Morphological analysis was carried out by three different methods: Fractal analysis (yellow area), Skeleton analysis (pink area) and Sholl analysis (grey area). Data distribution of each parameter is presented as violin plot, where the dashed line represents the median and the dotted line represents the quartiles. N=240-250 cells were sampled from different animals within each experimental group. Sal, saline; NA, neuraminidase; DMSO, dimethyl sulfoxide; PLX, PLX5622. *p < 0.05, **p < 0.01, ***p < 0.001.

## Slide 4
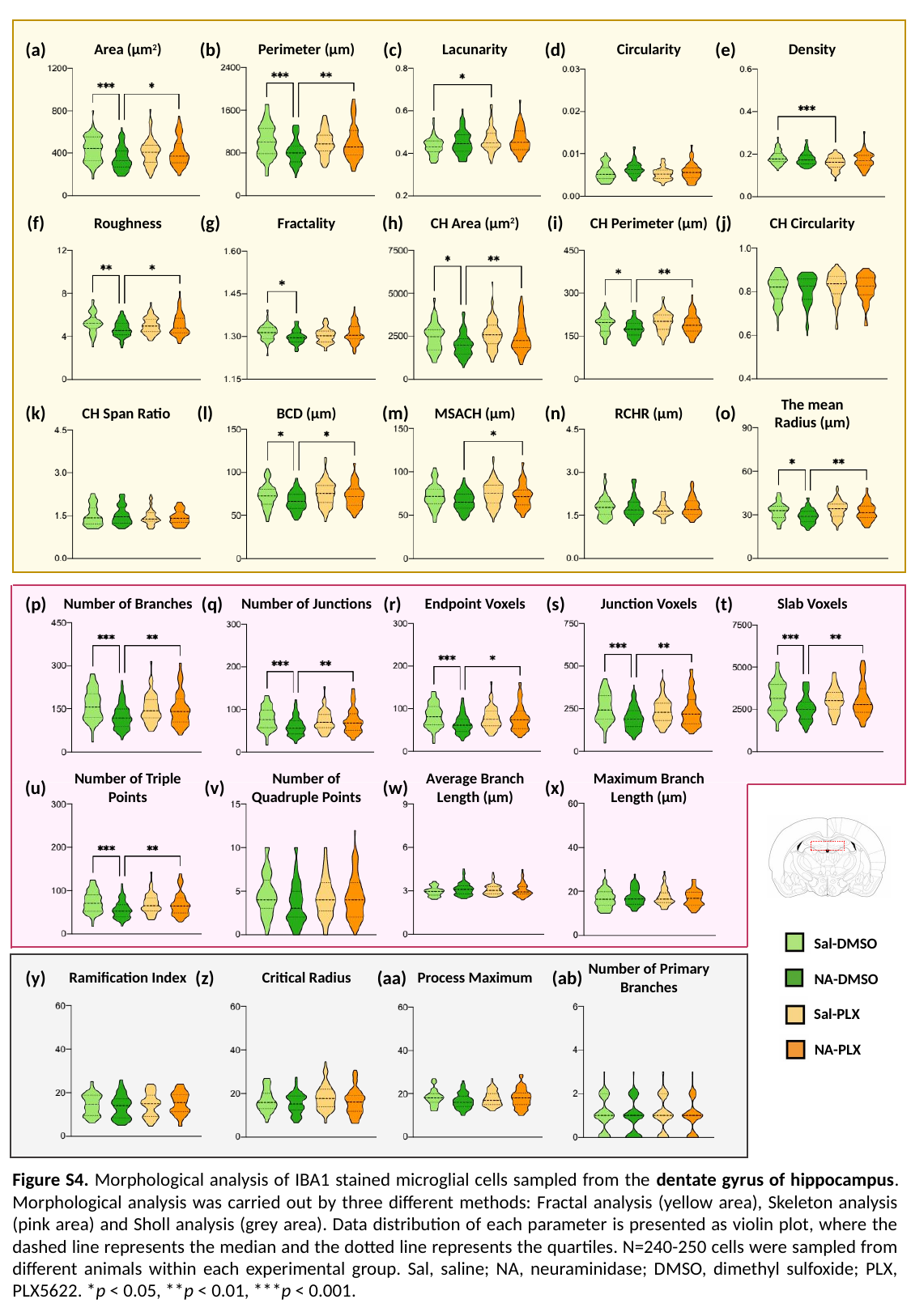

(a)
(b)
(c)
(d)
(e)
Area (μm2)
Perimeter (μm)
Lacunarity
Circularity
Density
(f)
(g)
(h)
(i)
(j)
Roughness
Fractality
CH Area (μm2)
CH Perimeter (μm)
CH Circularity
The mean Radius (μm)
(k)
(l)
(m)
(n)
(o)
CH Span Ratio
BCD (μm)
MSACH (μm)
RCHR (μm)
(p)
(q)
(r)
(s)
(t)
Number of Branches
Number of Junctions
Endpoint Voxels
Junction Voxels
Slab Voxels
Number of Triple Points
Number of Quadruple Points
Average Branch Length (μm)
Maximum Branch Length (μm)
(u)
(v)
(w)
(x)
Sal-DMSO
NA-DMSO
Sal-PLX
NA-PLX
Number of Primary Branches
(y)
(z)
(aa)
(ab)
Ramification Index
Critical Radius
Process Maximum
Figure S4. Morphological analysis of IBA1 stained microglial cells sampled from the dentate gyrus of hippocampus. Morphological analysis was carried out by three different methods: Fractal analysis (yellow area), Skeleton analysis (pink area) and Sholl analysis (grey area). Data distribution of each parameter is presented as violin plot, where the dashed line represents the median and the dotted line represents the quartiles. N=240-250 cells were sampled from different animals within each experimental group. Sal, saline; NA, neuraminidase; DMSO, dimethyl sulfoxide; PLX, PLX5622. *p < 0.05, **p < 0.01, ***p < 0.001.

## Slide 5
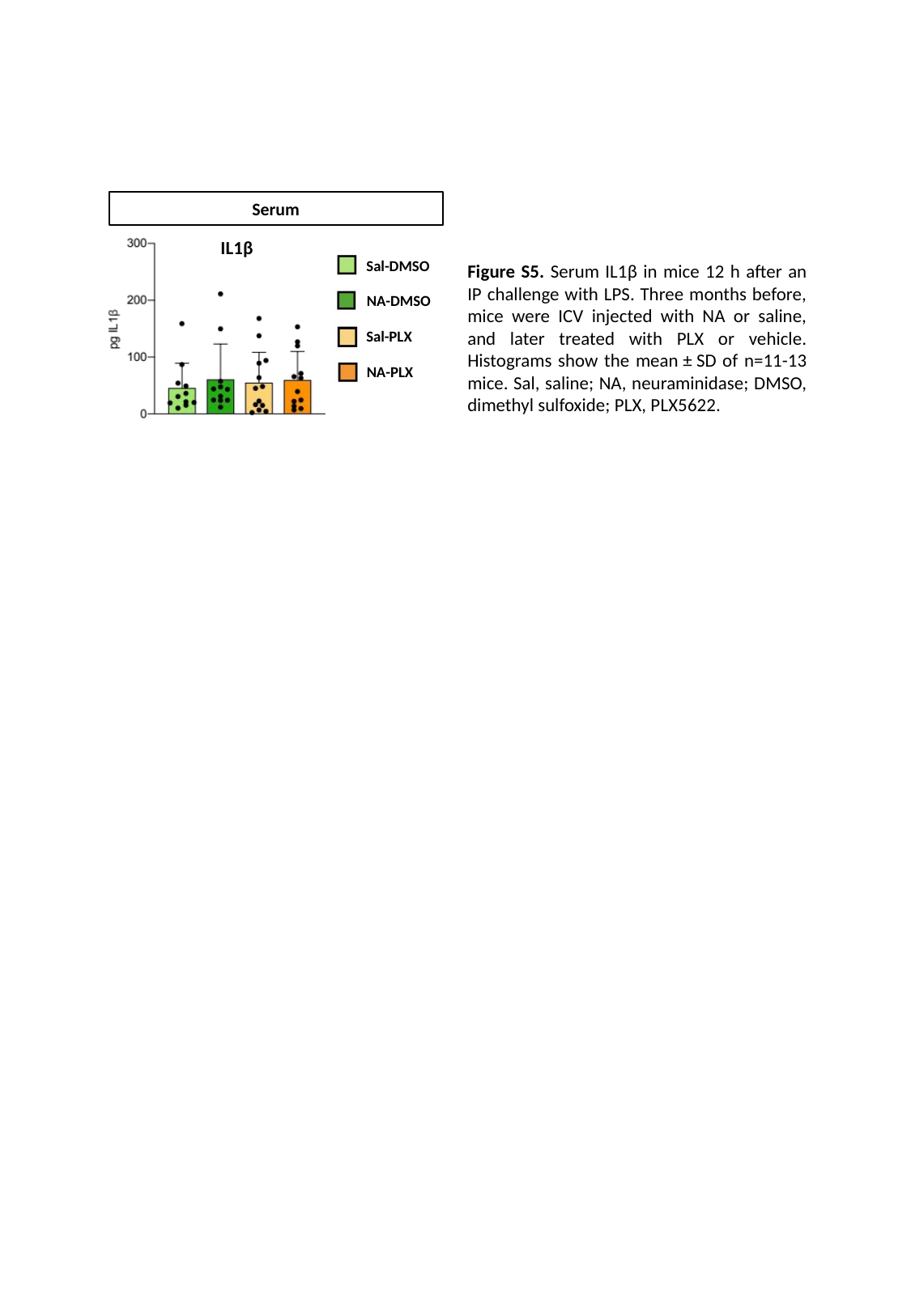

Serum
IL1β
Sal-DMSO
NA-DMSO
Sal-PLX
NA-PLX
Figure S5. Serum IL1β in mice 12 h after an IP challenge with LPS. Three months before, mice were ICV injected with NA or saline, and later treated with PLX or vehicle. Histograms show the mean ± SD of n=11-13 mice. Sal, saline; NA, neuraminidase; DMSO, dimethyl sulfoxide; PLX, PLX5622.
