# Supplementary material for: Long‐term reprogramming of primed microglia after moderate inhibition of CSF1R signaling
Source: Glia. 2024 Oct 24;73(1):175–95. doi: 10.1002/glia.24627 (PMC11660525; doi:10.1002/glia.24627)
Supplement: Supplementary file 2 — Table S1. Sequence of the primers used for qPCR. [file GLIA-73-175-s001.pptx]

## Slide 1
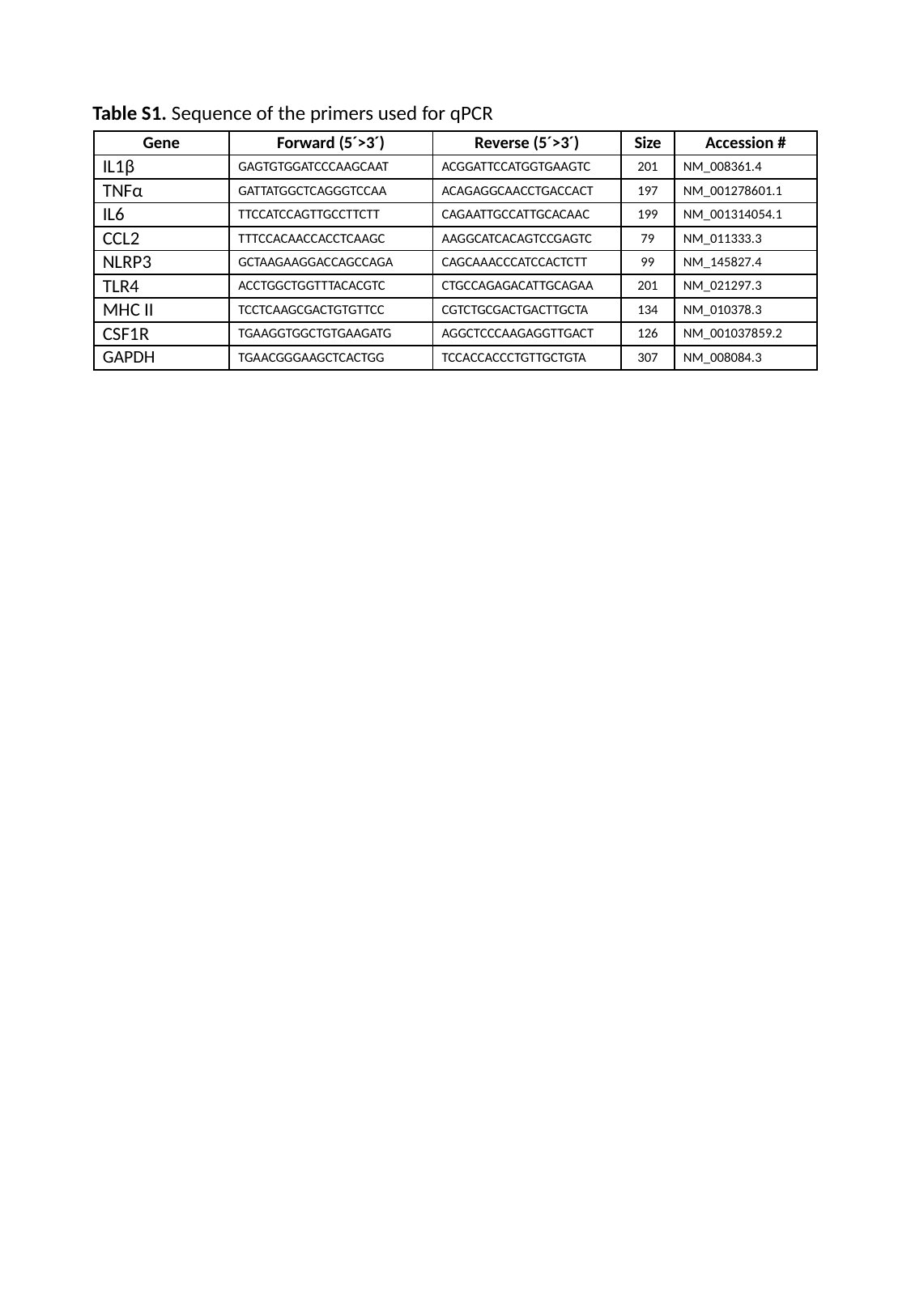

Table S1. Sequence of the primers used for qPCR
| Gene | Forward (5´>3´) | Reverse (5´>3´) | Size | Accession # |
| --- | --- | --- | --- | --- |
| IL1β | GAGTGTGGATCCCAAGCAAT | ACGGATTCCATGGTGAAGTC | 201 | NM\_008361.4 |
| TNFα | GATTATGGCTCAGGGTCCAA | ACAGAGGCAACCTGACCACT | 197 | NM\_001278601.1 |
| IL6 | TTCCATCCAGTTGCCTTCTT | CAGAATTGCCATTGCACAAC | 199 | NM\_001314054.1 |
| CCL2 | TTTCCACAACCACCTCAAGC | AAGGCATCACAGTCCGAGTC | 79 | NM\_011333.3 |
| NLRP3 | GCTAAGAAGGACCAGCCAGA | CAGCAAACCCATCCACTCTT | 99 | NM\_145827.4 |
| TLR4 | ACCTGGCTGGTTTACACGTC | CTGCCAGAGACATTGCAGAA | 201 | NM\_021297.3 |
| MHC II | TCCTCAAGCGACTGTGTTCC | CGTCTGCGACTGACTTGCTA | 134 | NM\_010378.3 |
| CSF1R | TGAAGGTGGCTGTGAAGATG | AGGCTCCCAAGAGGTTGACT | 126 | NM\_001037859.2 |
| GAPDH | TGAACGGGAAGCTCACTGG | TCCACCACCCTGTTGCTGTA | 307 | NM\_008084.3 |
